# Roles and mechanisms of garlic and its extracts on atherosclerosis: A review

**DOI:** 10.3389/fphar.2022.954938

**Published:** 2022-10-03

**Authors:** Min Li, Wingyan Yun, Guibin Wang, Anqi Li, Jing Gao, Qingyong He

**Affiliations:** ^1^ Department of Cardiology, Guang’anmen Hospital, China Academy of Chinese Medical Sciences, Beijing, China; ^2^ Clinical Department of Traditional Chinese Medicine, Beijing University of Chinese Medicine, Beijing, China

**Keywords:** garlic, *Allium sativum*, atherosclerosis, oxidative stress, inflammation, endothelial dysfunction

## Abstract

The prevention and treatment of cardiovascular diseases (CVDs) have achieved initial results, but the number of CVDs patients will increase rapidly in the next 10 years. Atherosclerosis (AS) is a significant risk factor for CVDs. The impact of lifestyle and daily diet varies considerably between different countries and continents and has been shown to affect the development of various diseases such as diabetes and CVDs. Primary and secondary prevention using alternative supplements and methods to avoid or reduce the use of traditional pharmacological drugs have also become popular. One of the reasons for this is that pharmacological drugs with lipid-lowering, and blood pressure-lowering effects cause many side effects that may negatively impact the quality of life. Patients are now emphasizing reliance on lifestyle changes to reduce cardiovascular risks. Garlic is a medicinal and edible plant that has been used for a long time. In order to reveal garlic application in the prevention and treatment of AS, reviewing the latest domestic and international studies through searching databases. The result shows that the antiatherogenic role of garlic is eximious. And the mechanisms are mainly related to hypolipidemic, antioxidant, antithrombotic, inhibiting angiogenesis, protecting endothelial cells, anti-inflammatory, anti-apoptotic, inhibiting vascular smooth muscle proliferation, and regulating gut microbiota. The main signaling pathways involve AMPK/TLRs, Keap1/Nrf2, PI3K/AKT, PPARγ/LXRα, GEF-H1/RhoA/Rac, etc. The antiatherogenic actions and molecular mechanism of garlic were reviewed in this study to obtain a robust evidence basis for the clinical application and mechanistic study and provide a theoretical basis for further utilization of garlic.

## Introduction

Atherosclerosis is a lipid-driven chronic inflammatory disease and increases morbidity and mortality of CVDs. Inflammation and abnormal lipid metabolism underlie the pathology of AS, mainly involving the inner and middle layers of arteries, resulting in thickening and stiffening of the arterial wall, narrowing of the lumen, and progressive loss of elasticity. Low-density lipoprotein (LDL) accumulates in blood vessels, causing monocytes to phagocytize. The foam cells formed by monocyte phagocytosis of LDL are prone to rupture. The released necrotic products stimulate the proliferation of fibrous tissue, forming fibrous plaques, which continue to develop into atheromatous plaques and eventually lead to AS.

CVDs are the leading cause of death from disease in China (Zhao et al., 2019) and are expected to cause 3 million deaths by 2030 (HALE Collaborators, 2017). The prevalence of CVDs in China is continuously rising, with an estimated 330 million people currently suffering from with CVDs (China Cardiovascular Health and Disease Report Writing Group, 2021). Lately, there has been increased interest and awareness in society regarding the connection between dietary intake and diseases. One of the reasons for this is that pharmacological drugs with lipid-lowering, and blood pressure-lowering effects cause many side effects that may negatively impact the quality of life.

Natural products, such as Chinese herbs, are an ideal source for developing safe and effective drugs for AS (Liu et al., 2015). Herbal or botanical preparations [complementary and alternative medicine (CAM)] have gained tremendous popularity in healthcare maintenance. A large number of populations in both developing and developed countries prefer to use CAM as a treatment and preventive measure for diseases (Qidwai et al., 2003; Frass et al., 2012). The 2007 National Health Interview Survey (NHIS) reported that approximately 38% of United States adults and 12% of children used CAM in the past 12 months; the usage rate of CAM has increased steadily all over the world since 1950 (Barnes et al., 2008; Omeish et al., 2011).

Garlic (*Allium sativum*) is the underground bulb of *Allium sativum* L., a member of the broad lily family. The hypolipidemic, antiatherogenic, anticoagulant, antidiabetic, antihypertensive, antimicrobial, anticancer, antioxidant, and immunomodulatory activities of garlic have been fully confirmed in basic and clinical research (HALE Collaborators, 2017). Aged garlic extract (AGE) inhibits coronary artery calcification progression, glucose levels, and blood pressure in patients at increased risk of cardiovascular events in a European cohort (Wlosinska et al., 2020). Phytochemical content in garlic could be a promising therapeutic agent in the future for the treatment of CVDs. Organosulfur compounds are the main active constituents of garlic, although the mechanism remains unclear (Gardner et al., 2007). In this study, the pharmacological effects of garlic and related mechanisms were reviewed to reveal its application in the prevention and treatment of AS.

## Materials and methods

Four databases of Chinese periodicals and three databases of English are searched comprehensively: China National Knowledge Infrastructure (CNKI, https://www.cnki.net/), Chinese Scientific Journals Full-Text Database (VIP, http://www.cqvip.com/), Wanfang Journal Database (WAN FANG, http://www.wanfangdata.com.cn/index.html) and China Biological Medicine Database (Sinomed, http://www.sinomed.ac.cn/), Pubmed (https://pubmed.ncbi.nlm.nih.gov/), Web of Science (http://isiknowledge.com), and Web of Science (https://www.webofscience.com). Studies on garlic for AS were screened from database construction to May 2022. The complete search strategy is attached (Supplementary Appendix A1). The search was conducted by two searchers using MeSH terms and entry terms. Two evaluators independently screened, evaluated, and cross-checked the literature according to the inclusion/exclusion criteria and consulted a third party to assist in discussing and resolving any disagreements.

### Inclusion criteria

The following types of studies were included: The following types of studies were included: 1) experimental studies; 2) clinical trials; 3) not a case report or a review; and 4) medicine identified as garlic or the garlic extract.

### Exclusion criteria

The following types of studies were excluded: The following types of studies were excluded: 1) full text not available; and 2) treatments combined with other ingredients.

## Results

**FIGURE 1:**
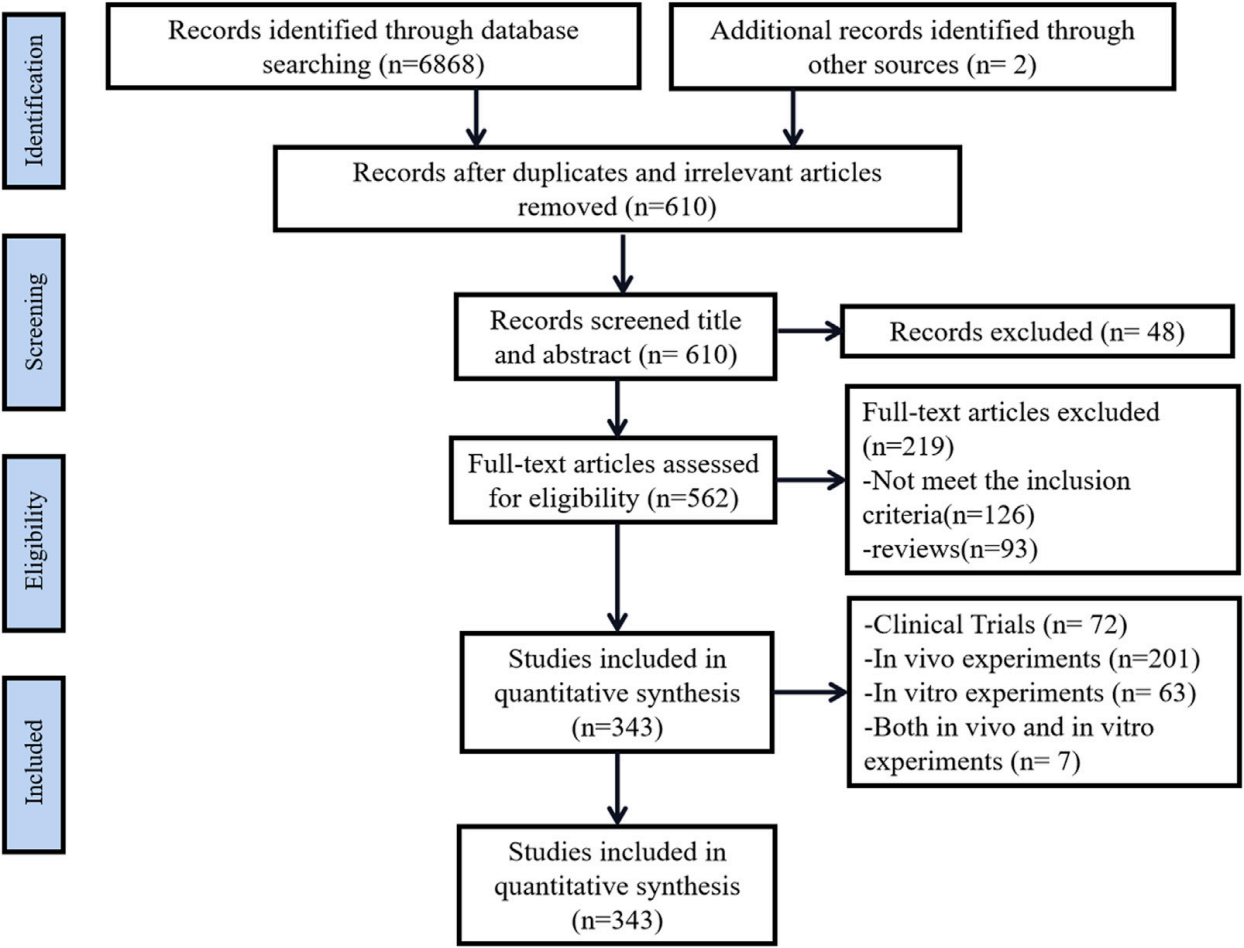
Summary of the literature search process.

Based on the screening results, 6870 studies were retrieved, and after excluding duplicate and irrelevant studies, 610 cases remained, and 48 unrelated studies were excluded after reading the titles and abstracts. 219 cases were excluded from reading the full text, of which 126 cases did not meet the inclusion criteria, 93 cases were comments, and finally, 343 cases were included, including 201 *in vivo* experiments, 63 *in vitro* experiments, seven *in vivo* experiments and *in vitro* experiments, and 72 clinical experiments. The retrieval process is shown in Figure 1.

## Botanical characteristics of garlic

**FIGURE 2:**
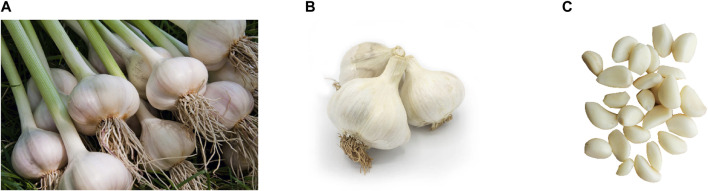
**(A)** Garlic plants. **(B)** Garlic bulbs. **(C)** Peeled garlic cloves.

Garlic is a biennial herb, that is, oblate or short conical in shape, with grayish or light brown membranous scale skin on the outside. Garlic has 6–10 cloves inside the bulbous leaves, borne in whorls around the flowering stem, with a disc-shaped stem base and numerous fibrous roots. Each garlic clove is covered with a membrane, peeled off to reveal white, thick, and juicy scales. It has a strong garlic odor and a pungent taste. Garlic leaves are solid, flat, linear-lanceolate, about 2.5 cm wide, and sheath-like at the base. The flowering stem is erect, about 60 cm high, the spathe has a long beak, 7–10 cm long, and the flowering period is 5–6 months. The bulbs are usually dug in spring and summer and used fresh or dried (Figure 2).

## History of garlic

Garlic is widely cultivated in Asia, Africa, and Europe (Tocmo et al., 2017). The Central Asian region is the origin of garlic. There are five species of garlic germplasm in the world, namely, Sativum, Ophioscorodon, Longicuspis, Subtropical, and Pekinense, of which Longicuspis is the most primitive, and other species are differentiated from it (Kamenetsky et al., 2005). Garlic was introduced to mainland China from Xinjiang during the Han Dynasty (Block, 1985). Xinjiang garlic is known as high alliin garlic because of its high active ingredients. The medicinal function of garlic can be traced back to 4000 years ago. At that time, garlic was used as an antiseptic and a stimulant, and the Chinese used garlic to treat exogenous fevers and headaches (Nagini, 2008). Consumption of fresh, cooked garlic is considered safe by the FDA (Rouf et al., 2020). Garlic is known for its wide range of biological activities, including anti-inflammatory (Xie and Du, 2011), antioxidant (Argüello-García et al., 2010; Elosta et al., 2017), anticancer (Petrovic et al., 2018), lipid-lowering (Brandolini et al., 2005), antihypertensive (Liperoti et al., 2017), antiatherogenic (Brown et al., 2015) and cardioprotective role (Bouillaud and Blachier, 2011). Studies have confirmed that garlic plays a role in preventive effects in cardiovascular and cerebrovascular diseases, tumors, diabetes, and others (Cavallito and Bailey, 1944). Some people have difficulty accepting garlic’s pungent and irritating taste but wish to consume garlic for long-term disease prevention and health care. Therefore, garlic products are innovated, and products such as garlic lyophilized powder, allicin, and garlic extract have been developed (Li et al., 2017).

## Chemical composition of garlic

**FIGURE 3:**
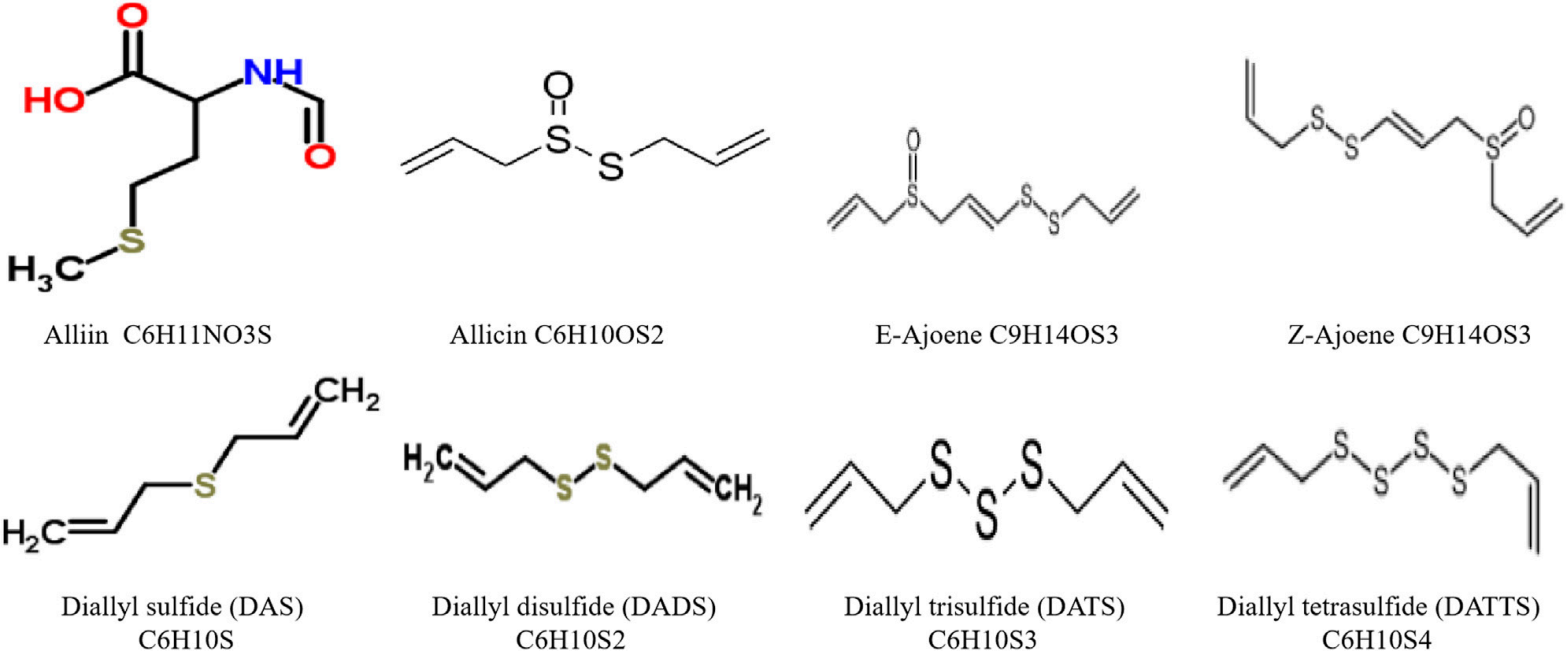
The representative sulfur compounds of garlic.

**FIGURE 4:**
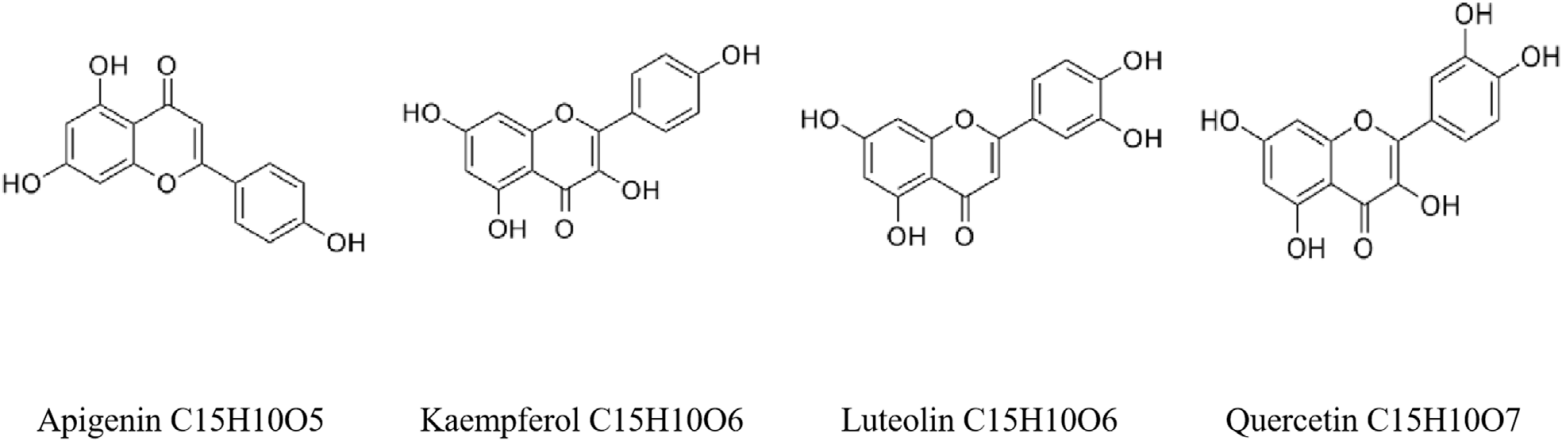
Other compounds of garlic.

More than 80 monomer compounds have been identified in garlic, mainly divided into volatile and nonvolatile compounds (Figures 3, 4).

### Volatile compounds

Volatile compounds in garlic include sulfur-containing compounds such as lipid-soluble organic sulfides and thiosulfinate (Hu et al., 2019). There are more than 30 kinds of sulfur-containing components in garlic, which is the primary bioactive substance of garlic. The main sulfur-containing compounds in garlic are alliin, allicin, etc. Alliin, chemically named S-Allyl-l-cysteine Sulfoxide (SACS), is an essential sulfur-containing compound in garlic bulbs. The most abundant sulfur-containing amino acid in garlic exists in the cytoplasm of garlic bulb cells (Almatroodi et al., 2019). Alliin inhibits platelet aggregation and may reduce the incidence of AS (Eric, 1985). Sheela et al. found that Alliin effectively reversed the elevation of lipids and lipid peroxides in hypercholesterolemic rats (Kendler, 1987). Alliinase and allinase are endogenous enzymes in garlic. Allinase, also known as Alliin lyase, is a dimeric glycoprotein in the vacuole of garlic scale bud cells and is more sensitive to temperature (Weiner et al., 2009). Allinase activity decreases when garlic is heated and vinegared.

Studies have shown that garlic has played a significant role in medicinal and edible due to the various thiosulfinate. The primary efficacy component is diallyl thiosulfinate (allicin) (Cavallito and Bailey, 1944). Thiosulfinate can inhibit the growth and reproduction of many germs and achieve better roles in anti-inflammatory and sterilizing. It also has antitumor (Bat-Chen et al., 2010), antioxidant (Ilić et al., 2015), lipid-lowering (Elkayam et al., 2013), and glucose-lowering (Arellano-Buendía et al., 2020) effects. After garlic is cut or crushed, alliin contained in the cell meets allinase and splits to produce allicin (Lanzotti, 2006). Allicin is unstable due to its sulfoxide and allyl structure and can be decomposed in a few hours at room temperature in the air. Allicin is the main component of fresh garlic homogenate (Salehi et al., 2019). Allicin can be further decomposed to produce the more stable ajone, dallyl sulfide (DAS), dallyl disulfide (DADS), and a small amount of dallyl trisulfide (DATS), dallyl Tetrasulfide (DATTS), which is the main component of new garlic oil extracted by steam distillation (Cao and Chen, 2008).

Since allicin is unstable, it cannot be prepared directly for use as a medicine. Allicin’s production rate is low because gastric acid inactivates allinase when consuming raw garlic. Therefore, to ensure the stability of the active ingredients of garlic, low-temperature extraction, microwave inactivation, chromatographic separation, ultrafiltration purification, spray drying, and other methods are often used to prepare new garlic preparations with high efficiency. At present, most companies at home and abroad make lyophilized garlic powder and enteric formulations of lyophilized garlic powder to improve the production rate of allicin *in vivo* (Yang et al., 2021).

DATS is known as a natural broad-spectrum antibiotic. DATS crosses the blood-brain barrier, scavenges free radicals, and achieves antioxidant effects by reducing the production of reactive oxygen species (Yan and Zeng, 2004). DATS has anti-mutagenic and anti-carcinogenic effects (Hong et al., 1992). Methyl allyl trisulfide (MATS) is the most substantial antifungal component in essential garlic oil, which can inhibit platelet aggregation and prevent thromboxane synthesis (Ariga et al., 1981). Ajone was reported as an active substance with a solid antiplatelet aggregation effect (Block, 1985, 1986). Ajone plays an essential role in cell-mediated immunity, humoral immune regulation, and other processes (Lawson et al., 1991).

### Nonvolatile compounds

Another class of compounds in garlic is nonvolatile compounds, mainly including water-soluble organic sulfides, steroidal saponins, saponin elements, flavonoids, phenols, peptides, enzymes, and organoselenium, organogermanium, hemagglutinin, fructans, prostaglandins, etc.

Up to now, 20 saponins have been extracted, isolated, and identified from garlic (Matsuura, 2001). Two new steroidal saponins were isolated from the water-soluble fraction of garlic, one was Proto-iso-eruboside B, and the other was iso-eruboside B. In addition, there were eruboside B and Sativoside C. Further, Sativoside -B2, -B3, -B4, and -B5 could be isolated from eruboside-B (Peng et al., 1996). Spirostanol saponin and Furostanol saponin are the main saponin components of garlic, and both saponins have hypolipidemic, antibacterial, and antitumor effects (Yan and Zeng, 2004). Garlic saponin can significantly inhibit platelet aggregation and prolong blood coagulation time to prevent and delay thrombosis.

Additionally, it can promote fibrinolysis (Peng et al., 1996). Garlic polysaccharide is one of the active ingredients with high content in garlic, accounting for 51% of the dry weight of garlic. Garlic polysaccharide with higher concentration has a more vital scavenging ability for free radicals (Lee et al., 2015). Six flavonoids and two polyphenols have been isolated from garlic, including apigenin, kaempferol, quercetin, luteolin, and N-ferulic acid-based tyramine.

In addition, in garlic, proteins, vitamins, fatty acids, biotin, nicotinic acid, and rare elements such as selenium and germanium have prominent pharmacological activities, such as antibacterial and anti-inflammatory, and cholesterol reduction.

## The pharmacological activity of garlic

### Hypolipidemic

Lipid migration is essential in developing AS (Wu et al., 2018). Oxidative modification of LDL induces phagocytosis and conversion of macrophages to foam cells (Wang H et al., 2017; Sharifi et al., 2019). Lecithin cholesterol acyltransferase (LCAT) catalyzes the esterification of free cholesterol, which promotes the maturation of high-density lipoprotein (HDL) and the reverse transport of cholesterol. Lipoprotein lipase (LPL) is the main enzyme in plasma that catalyzes the esterification of triglyceride-rich lipoproteins. Hepatic lipase (HL) can encourage the liver to take up and remove triglyceride-rich lipoprotein residues. The increased activity of these enzymes will stimulate the metabolism and transformation of lipids and reduce the level of blood lipids.

Kwak et al. (2014) found a reduction in serum total cholesterol (TC) levels in patients with hypercholesterolemia after taking garlic powder by Meta-analysis. Matsumoto et al. (2016) conducted a prospective randomized, double-blind trial of garlic extract for metabolic syndrome. They found that plaque reduction was significantly more significant in the AGE group than in a placebo group and that AGE also could stabilize vulnerable plaque. A meta-analysis of garlic modulation of serum TC conducted by Ried (2016) showed that garlic effectively reduced TC and Low-Density Lipoprotein Cholesterol (LDL-C) concentrations and increased macrophage activity and T and B cell production, with immunomodulatory effects.

Garlic oil could reduce TC and Triglyceride (TG) concentrations in hyperlipidemic mice, probably by promoting the metabolism and conversion of lipoproteins, or inhibiting intestinal cholesterol absorption, slowing down hepatic cholesterol synthesis, and thus accelerating TG breakdown (Zhang et al., 2007; Meng et al., 2016). The expression of Scavenger receptor class A (SR-A) and the cluster of differentiation 36 (CD36) directly affects the lipid deposition in macrophages and has an essential impact on macrophage foaminess (Twigg et al., 2012). C-Jun N-terminal kinase (JNK) and p38 mitogen-activated protein kinase (p38 MAPK) of mitogen-activated protein kinase (MAPK) pathway are critical signal pathways for regulating scavenger receptors and AS (Nikolic et al., 2011). JNK has been reported to regulate AS by regulating scavenger receptor expression, and JNK knockdown macrophages inhibit lipid uptake and foam formation (Sumara et al., 2005; de Nigris et al., 2012). Allicin could significantly inhibit the activation of JNK and p38 MAPK and down-regulate the increase of SR-A and CD36 expression, suggesting that allicin affects macrophage foam formation by regulating the expression of scavenger receptors (Wang L et al., 2017). Allicin promotes cholesterol efflux through upregulation of ATP Binding Cassette Subfamily A Member 1 (ABCA1). It reduces lipid accumulation in THP-1 macrophage-derived foam cells by activating Peroxisome proliferator-activated receptor Gamma (PPARγ)/Liver X receptor α (LXRα) signaling (Lin et al., 2017).

Inhibition of cholesterol synthesis by S-allyl cysteine (SAC), S-ethyl cysteine (SEC), and S-propyl cysteine (SPC) was achieved by inhibiting the activity of hydroxy-3-methyl glutaryl coenzyme A reductase (HMGCR) (Liu and Yeh, 2002). N-acetylcysteine (NAC), SEC, and SPC reduce TG and TC accumulation in the liver by a mechanism associated with the reduction of sterol regulatory element-binding protein-1c (SREBP-1c), sterol regulatory element-binding protein-2 (SREBP-2) (Lin and Yin, 2008). DADS inhibits hepatic TG and relates lipid synthesis by downregulating SREBP-1c expression. In addition, DADS can accelerate TG catabolism and fatty acid oxidative metabolism by upregulating the expression of peroxisome proliferator-activated receptor α (PPARα) in hepatocytes (Wang et al., 2019). SAC activates AMP-activated protein kinase (AMPK) through calcium/calmodulin-dependent kinase (CaMKK), silent information regulator T1, and inhibits sterol regulatory element-binding proteins-1 (SREBP-1)-mediated hepatic adipogenesis (Hwang et al., 2013). Allicin was found to enhance the activity of serum LCAT, LPL, and HL, thereby reducing plasma TG and TC levels (Zhang et al., 2007).

Microsomal triglyceride transfer protein (MTP) is the rate-limiting factor for VLDL apoB secretion. MTP plays an essential role in regulating lipoprotein production in the liver and intestine. Inhibition of MTP activity has been shown to decrease the rate of secretion of apoB-containing lipoproteins in HepG2 cells (Jamil et al., 1996) and intestinal Caco-2 cells (van Greevenbroek et al., 1998) *in vitro*. Fresh garlic extract (FGE) inhibits the synthesis and secretion of intestinal chylomicrons by suppressing the expression of microsomal MTP genes for lipid-lowering (Lin et al., 2002). Acyl-CoA: cholesterol acyltransferase (ACAT) is the rate-limiting enzyme that catalyzes the formation of cholesteryl esters by linking cholesterol with long-chain fatty acids (Yao et al., 2006). Garlic powder extract (GPE) was able to inhibit the activity of ACAT and enhance the activity of Cholesteryl ester hydrolase (CEH) (Orekhov and Tertov, 1997), thus significantly reducing the accumulation of intracellular cholesteryl esters.

### Antioxidant

Oxidative stress (OS) is mainly caused by the increase of reactive oxygen species (ROS) and reactive nitrogen species (RNS), which can lead to endothelial remodeling, tissue damage, and eventually AS (Canugovi et al., 2019). In endothelial cells (ECs), vascular smooth muscle cells (VSMCs), and macrophages, mitochondrial dysfunction and nicotinamide adenine dinucleotide phosphate oxidase (NOX) can cause excessive ROS production *in vivo* and accelerate the development of AS by causing the impaired function of ECs, proliferation, and migration of VSMCs, macrophage foaminess and inflammatory response through various pathways.

A meta-analysis shows that garlic supplementation improves antioxidant status by increasing total antioxidant capacity (T-AOC) and decreasing Malondialdehyde (MDA) levels (Moosavian et al., 2020). SAC inhibits Oxidized Low-Density Lipoprotein (ox-LDL) (Ide and Lau, 2001), inducible nitric oxide synthase (iNOS) activity (Kim et al., 2001), peroxides production, and prevents intracellular glutathione (GSH) depletion in ECs. In addition, it has been shown that garlic polysaccharides can reduce MDA, Nitric Oxide (NO) levels in serum and inhibit lipid peroxidation in mice (Li et al., 2003). DADS activates the Kelch Like ECH Associated Protein 1 (Keap1)/nuclear erythroid 2-related factor 2 (Nrf2) pathway and upregulates NAD. (P)H: quinone oxidoreductase 1 (NQO1), γ-glutamylcysteine synthetase antibody (γ-GCSc), and Superoxide Dismutase 1 (SOD-1) expression levels, and improves hepatic oxidative stress levels in high-fat-fed Wistar rats (Wang et al., 2019). The up-regulation of Sirtuin 1 (SIRT1) activity can reduce ROS production in endothelial cells, thus protecting vascular endothelial cells from ROS damage (Wu et al., 2014). Allicin can promote the phosphorylation of SIRT1, upregulate the activity of SIRT1, and reduce the production of ROS and the expression of plasma activator inhibitor-1 (PAI-1) in cells (Suo et al., 2013; Hu et al., 2016). Garlic sulfide can regulate mitochondrial respiration in cardiac myocytes and produce hydrogen sulfide *via* myocardial mitochondria, which can diastole contracted vascular smooth muscle (Li et al., 2016) and play the anti-myocardial ischemic effect. Total saponins of garlic (TSG) exerted antioxidant effects by inhibiting MDA content and restoring reduced superoxide dismutase (SOD) activity (Miao et al., 2020). Activation of ox-LDL and nuclear factor kappa-B (NF-κB) is associated with AS, and SAC inhibits ox-LDL activation in macrophages and human umbilical vein endothelial cells (HUVECs) of J774 mice with dose-dependent inhibition of NF-κB activation. It indicated that SAC could slow the progression of AS by inhibiting ox-LDL and interfering with the cascade of oxidative signaling (Ho et al., 2001). Treatment of New Zealand rabbits receiving a high-cholesterol diet with allicin significantly reduces MDA while increasing GSH and SOD levels (El-Sheakh et al., 2016). The antiatherogenic effect of allicin may be related to its ability to scavenge free radicals and restore antioxidant defense systems.

### Antithrombotic

During plaque formation, changes in hemodynamics increase plaque instability and can easily lead to plaque rupture. Once the plaque ruptures, platelet activation in the blood, activation of the coagulation pathway cascade, and multicellular mobilization lead to platelet-rich emboli that block blood vessels and cause malignant clinical events such as ischemic myocardial infarction and stroke (Kobiyama and Ley, 2018). Antithrombotic therapy is an essential part of the treatment of patients with AS. Activated platelets mediate plaque instability involved in chronic AS development by releasing large amounts of inflammatory secretions and expressing multiple membrane immune receptors interacting with different leukocyte subpopulations and endothelial cells (Siegel-Axel et al., 2008; Peter Seizer, 2008; Li et al., 2011).

Possible mechanisms for the antithrombotic activity of garlic include: inhibiting the secretion of Cyclooxygenase-1 (COX1), reducing the synthesis of Thromboxane B2 (TXB2), reducing the secretion of Leukotriene C4 (LTC4C4C) and Prostaglandin E2 (PGE2) (Bordia et al., 1996), reducing the release of arachidonic acid (AA) (Mohammad and Woodward, 1986) from phospholipids, upregulating 5-hydroxy tryptamine (5-HT) and inhibiting the release of coagulation factor IV from platelets (Makheja and Bailey, 1990). Garlic effectively inhibits platelet aggregation induced by the calcium ion aggregate A23187. Therefore, the antiplatelet aggregation effect of garlic may be related to the mobilization of calcium within platelets (Srivastava, 1986).

A double-blind placebo-controlled randomized study showed that treating patients with cerebral atherosclerosis with garlic powder pills (Allicor) for 14 days resulted in a 25% reduction in ADP-induced platelet aggregation and a 22% upregulation in plasma fibrinolytic activity (Sobenin et al., 2010). However, a randomized, double-blind placebo-controlled crossover study showed that garlic oil caused a significant 12% reduction in epinephrine-induced platelet aggregation but had no effect on collagen-induced or ADP-induced platelet aggregation (Wojcikowski et al., 2007). This contradiction suggests that garlic may inhibit platelet aggregation through multiple mechanisms. Clinical studies have confirmed that processed garlic improves the potency and bioavailability of organosulfides and is more likely to exert antithrombotic effects than raw garlic (Lawson and Gardner, 2005). A randomized controlled trial confirmed that garlic oil significantly increased fibrinolytic activity. The standard components of garlic oil, DADS and DATS, inhibited platelet agonist-induced platelet aggregation (PAg) and platelet thromboxane formation (Bordia et al., 1998).

TXB2 and prostaglandin I2 (PGI2) are metabolites of AA. Thromboxane A2 (TXA2) induces vasoconstriction and platelet aggregation and promotes AS formation. In contrast, PGI2 has the function of vasodilation and inhibiting platelet aggregation. The TXA2 and PGI2 are usually determined by measuring their stable metabolites TXB2 and 6-keto-prostaglandin F1α (6-keto-PGF1α). Total saponins of garlic (TSG) reduces the level of TXB2 and increases the level of 6-keto-PGF1α, and the ratio of TXB2 to 6-keto-PGF1α is maintained in a relatively stable dynamic equilibrium to maintain vascular homeostasis (Miao et al., 2020). α-Granules and dense granules enhanced the conduction pathway of platelet activation. A rapid release of ATP characterizes the early phase of platelet activation. Fermented and non-fermented garlic products inhibit ATP release from dense granules and exert antiplatelet effects by inhibiting platelet granule secretion (Irfan et al., 2019). Adhesion molecules such as Vascular Cell Adhesion Molecule 1 (VCAM-1) and intercellular cell adhesion molecule-1 (ICAM-1) promote platelet adhesion and leukocyte recruitment and play an essential role in AS formation (Kim et al., 2018). Garlic powder decreased VCAM-1and ICAM-1 level, significantly increasing activated partial thromboplasting time (APTT). It indicated that garlic might contribute to treating AS by delaying clotting time, altering angiotensin, and decreasing the expression of VCAM-1 and ICAM-1.

Tissue factor (TF) is a crucial factor in initiating the extrinsic coagulation cascade pathway, which is responsible for producing thrombin from prothrombin *via* activation of factor VII (Dahlbäck, 2000). Subendothelial TF is also accountable for initiating fibrin formation at sites of vascular injury, and blood-borne TF may be a vital contributor to the propagation of the developing thrombus (Grover and Mackman, 2020). It has been reported that tumor necrosis factor-α (TNF-α) induced TF mRNA expression in HUVECs was suppressed by the inhibition of JNK. Therefore, inhibition of the JNK pathway by DATS may inhibit the induction of TF by TNF-α. DATS inhibited not only TF activity but also TF mRNA and protein expression *in vitro* (Okue et al., 2022). Garlic is a promising food with anti-thrombotic function, which can suppress both primary and secondary clot formation. The antithrombotic effect of garlic is beneficial for patients who are allergic or intolerant to aspirin and is expected to be an alternative or complementary therapy to antiplatelet therapy.

### Inhibit angiogenesis

Plaque angiogenesis is considered to play an essential role in the pathophysiological development of AS. Neointimal angiogenesis is highly related to plaque formation and the risk of plaque rupture. Plaque angiogenesis quickly leads to the formation of plaque and thus increasing the risk of rupture (Sluimer and Daemen, 2009). In the past 30 years, the research mainly focused on the role of new blood vessels in plaque formation and rupture (Kockx et al., 2003; Sluimer et al., 2009), which revealed that there was an expanding network of new blood vessels in plaque in the stenosis near the inflammatory infiltration and necrotic core. Plaque angiogenesis is related to plaque vulnerability and plaque erosion. Many angiogenic factors participate in plaque formation, mainly vascular endothelial growth factor (VEGF) and basic fibroblast growth factor (bFGF).

Akt activation is associated with angiogenesis (Morales-Ruiz et al., 2000). *In vitro* and *in vitro* experiments proved that Allicin can inhibit angiogenesis and weaken epithelial cell proliferation, tubule formation, actin polymerization, and Akt phosphorylation by reducing VEGF and bFGF expression (Sela et al., 2008). DATS is an effective inhibitor of the angiogenic properties of HUVECs *in vitro*, for example, inhabiting capillary tube formation and migration. It is related to caspase-dependent induction of apoptosis, inhibition of VEGF secretion, inactivation of vascular endothelial growth factor receptor 2 (VEGF-R2) and Akt kinase, and activation of extracellular regulated protein kinases (ERK1/2) (Xiao et al., 2006).

### Endothelium protection

ECs dysfunction is a crucial factor in the development of AS (Donato et al., 2018). The dysfunction of ECs refers to a decrease in NO-mediated vasodilatory responses in ECs in response to different pathological stimuli. The excessive synthesis of Endothelin-1 (ET1) leads to an increase in vasoconstriction and vascular permeability. This change can lead to the release of pro-inflammatory factors, over-activation of platelets, enhanced oxidation of LDL, and proliferation and migration of vascular smooth muscle cells (Gimbrone and García-Cardeña, 2016; Pi et al., 2018).

Clinical studies have found that allicin reduces ET-1 and C-reaction protein (CRP) levels and elevates NO levels, improves endothelial dysfunction, and reduces the incidence of restenosis in patients after PCI (Wang and Fu, 2009). In patients with coronary artery disease combined with diabetes, oral administration of allicin capsules resulted in a significant improvement in flow-mediated dilation (FMD) and NO levels, and a decline in ICAM-1 level and incidence of major adverse cardiovascular events in the allicin group compared to the control group, which may be related to allicin’s improvement in endothelial function (Nie et al., 2013). A randomized, placebo-controlled, crossover trial suggested that short-term treatment with AGE may improve endothelial function in patients with coronary artery heart disease (CHD) (Williams et al., 2005). Another randomized, double-blind, placebo-controlled trial found that AGE supplementation was beneficial in reducing endothelial biomarkers associated with cardiovascular risks, such as the arterial stiffness index (SI), high-sensitivity C-reactive protein (hsCRP), PAI-1 as well as total antioxidant status (TAS) (Szulińska et al., 2018).

The adhesion of leukocytes/monocytes to endothelium is an early event of AS. Garlic extracts significantly reduce the expression of ICAM-1 and VCAM-1 induced by Interleukin-1 alpha (IL-1a) and considerably inhibit the adhesion of monocytes to endothelial cells stimulated by IL-1a (Rassoul et al., 2006). l-arginine in AGE promotes NO production mediated by Endothelin nitric oxide synthase (eNOS), leading to vasodilation (Nie et al., 2002; Takashima et al., 2017). Oral DADS analogs can reverse L-N G-Nitro arginine methyl ester (l-NAME)-induced systolic blood pressure, oxidative stress, Angiotensin Converting Enzyme (ACE) activity, cyclic guanosine monophosphate (cGMP), and NO levels, which may be related to activation of eNOS (Williams et al., 2005). DAT significantly reduced the levels of MDA and ROS in mitochondria and increased the activities of SOD and Glutathione peroxidase (GSH-Px). DAT protects the vascular endothelium from hyperglycemia-induced damage by reducing oxidative stress in mitochondria (Liu et al., 2014). The calcium-sensing receptor (CaSR) is a member of the G protein-coupled receptor superfamily, and activation of CaSR reduces cell viability and promotes apoptosis (Zhang et al., 2020). Allicin may inhibit cardiomyocyte apoptosis and protect vascular endothelial function by suppressing the expression of CaSR and inhibiting the oxidative stress response (Xu et al., 2020). This suggests that garlic consumption may reduce oxidative damage to endothelial cells and improve vascular function.

Vascular endothelial barrier function is maintained by a cell-to-cell junctional proteins and contributes to vascular homeostasis (Chistiakov et al., 2015). Various risk factors such as inflammation disrupt barrier function through down-regulation of these proteins and promote vascular diseases such as atherosclerosis (Hofmann et al., 2002).

AGE and its primary sulfur-containing constituent, S-1-propenylcysteine (S1PC), reduced hyperpermeability elicited by TNF-α in HUVECs. In addition, S1PC inhibited TNF-α-induced production of myosin light chain (MLC) kinase and inactivation of MLC phosphatase through the suppression of the Rac and Ras homolog gene family, member A (RhoA) signaling pathways, respectively, which resulted in the dephosphorylation of MLC2, a key factor of actin remodeling. Moreover, S1PC inhibited the phosphorylation and activation of guanine nucleotide exchange factor-H1 (GEF-H1), a common upstream key molecule and activator of Rac and RhoA. These effects of S1PC were accompanied by its ability to prevent the disruption of junctional proteins in the cell-cell contact regions and the increase of actin stress fibers induced by TNF-α (Kunimura et al., 2021). The study suggested that AGE and S1PC improve endothelial barrier disruption by inhibiting the GEF-H1/RhoA/Rac pathway.

### Anti-inflammatory

It is generally known that AS is considered a chronic inflammatory disease because inflammation goes through all AS processes and plays an essential role. When ECs are activated, Inflammatory factors such as monocyte chemotactic protein 1 (MCP-1), interleukin-8 (IL-8), ICAM-1, VCAM-1, Endothelial leukocyte adhesion molecule-1 (ELAM-1), and granular membrane protein 140 (GMP140) attract lymphocytes and monocytes bound to ECs and arterial walls, contributing to inflammation (Zhu et al., 2018). In addition, monocytes differentiate into macrophages which can phagocytize ox-LDL and ultimately transform it into lipid-laden foam cells (Kattoor et al., 2019; Javadifar et al., 2021). During arterial endothelium damage, foam cells form and accumulate and release inflammatory mediators such as MCP-1 and TNF-α (Javadifar et al., 2021). The activation of the NF-κB signaling pathway stimulates the formation of the inflammatory process, which leads ECs to take on AS phenotypes in the carotid sinus (Tabas et al., 2015). In a randomized, double-blind placebo-controlled trial by Martiné Wlosinska et al., 104 AS patients took 2400 mg AGE capsules daily for 12 months. The results showed that AGE could effectively decrease levels of IL-6 (Wlosinska et al., 2020). AGE’s anti-atherosclerosis effects include reducing CRP and TXB2, down-regulating TNF-α and interleukin-1 receptor-activated kinase 4 (IRAK4) productions, and increasing AMPK activity in the liver (Morihara et al., 2017). AGE also regulates the inflammatory process by inducing AMPK activation and down-regulating the Toll-like receptor (TLR) signaling pathway (Miki et al., 2017). Z-Ajone reduces the phosphorylation and nuclear translocation of STAT3, inhibits the activity of Cyclooxygenase-2 (COX2) (Hitchcock et al., 2021), and carries out S-sulfhydrylation of cysteine sulfhydryl groups in two inflammatory proteins, thus producing downstream anti-inflammatory effects.

The innate and adaptive immunity cells play a significant role in atherosclerosis progression. Dynamic change in blood lipid levels could trigger CD4^+^ T-cells to differentiate into effector cells and produce cytokine during atherogenesis (Tabas and Lichtman, 2017). T helper 1 cell (Th1) is the subset of T lymphocytes mostly found in atherosclerotic lesions based on the cytokine it produces. Th1 cells secrete Interferon-gamma (IFN-g) and TNF-a proinflammatory cytokines to enhance immune response through macrophage activation, smooth muscle cells, and endothelial cells during atherogenesis (Wu et al., 2017). A study confirmed that Single garlic oil could suppress CD4 t-cells activation and NF-κB expression in high-fat diet mice. Furthermore, Single garlic oil plays a role as an athero-protective agent in the High-fat diet condition through the decrease in proinflammatory cytokines such as TNF-a and IFN-g (Lestari et al., 2020). SGO could act as a promising prospect for therapy to improve chronic inflammation in AS.

It has been reported that the development of atherosclerosis alters the ratio of polarized macrophages. M1 macrophages promote the formation of AS plaques by sustaining inflammation, whereas M2 macrophages aid the regression of atherosclerotic (Yang et al., 2020). AGE increased the mRNA or protein levels of arginase1 (Arg1), interleukin-10 (IL-10), CD206, and hypoxia-inducible factor 2α (HIF2α). It decreased that of CD68, HIF1α, and inducible NO synthase in the aorta and spleen of Apo E^−/−^ mice. S1PC increased the level of IL-10-induced Arg1 mRNA and the extent of M2c-like macrophage polarization *in vitro*. In addition, S1PC increased the population of M2c-like macrophages, suppressing the people of M1-like macrophages and decreasing lipopolysaccharide-induced production of pro-inflammatory cytokines. These effects were accompanied by prolonged phosphorylation of the IL-10 receptor α (IL-10Rα) and signal transducer and activator of transcription 3 (STAT3) that inhibited the interaction between IL-10Rα and Src homology-2-containing inositol 5′-phosphatase 1 (SHIP1) (Miki et al., 2021). These findings suggest that S1PC may help improve atherosclerosis due to its anti-inflammatory effect in promoting IL-10-induced M2c macrophage polarization.

### Anti-apoptotic

Cell proliferation and apoptosis rates are key indicators of cell viability and apoptosis, further aggravating atherosclerotic plaque’s progression and instability. Allicin significantly increased the cell viability of HUVECs, inhibited apoptosis, and protected against ox-LDL-induced damage in HUVECs by inhibiting caspase-3 and Nicotinamide adenine dinucleotide phosphate (NADPH) oxidase-related apoptotic signaling pathways (Chen et al., 2016).

H2O2 can cause apoptosis in vascular ECs through multiple pathways and is recognized as a standard model of oxidative injury. Allicin effectively reduced H2O2-induced apoptosis in HUVECs, probably because allicin stabilized the expression of pro-Caspase-3 protein and decreased the expression of poly adenosine diphosphate-ribose polymerase (PARP) and BCL2-Associated X (Bax) proteins. Allicin can increase SOD, NO, and eNOS and decrease MDA, indicating that allicin can protect HUVECs induced by H_2_O_2_ from apoptosis by reducing oxidative stress (Chen et al., 2014).

Allicin can attenuate apoptosis induced by Lipopolysaccharide (LPS), and its mechanism is related to inhibition of mitochondrial dysfunction, such as inhibition of matrix metalloproteinases (MMPs) collapse, reduction of cytochrome c synthesis, and mitochondrial ATP release (Zhang et al., 2017). Cardiomyocyte apoptosis plays a vital role in the development of AS. High cholesterol diet-induced apoptosis in cardiomyocytes is associated with Fatty acid synthase (Fas)-dependent and mitochondria-dependent apoptotic pathway activity. Mitochondrial-dependent pathway plays an essential role in cell apoptosis by releasing caspase 9. Garlic activates the PI3K-Akt pathway, inhibits TNF-α, Fas, caspase 8, caspase 9, and caspase 3, upregulates the protein level of mitochondrial B-cell lymphoma-2 (Bcl-2), and reduces the protein levels of recombinant human bh3-interacting domain death agonist (B.I.D.) and Bax (Cheng et al., 2013), thus inhibiting myocardial cell apoptosis.

### Other antiatherogenic mechanisms

In addition to the above mechanisms, garlic and its extracts can also inhibit the development of AS by inhibiting the proliferation of vascular smooth muscle, regulating gut microbiota and supplement with phytoestrogens.

An essential feature of AS is the transformation of quiescent or differentiated VSMCs into proliferating or dedifferentiated cells, leading to enhanced migration of VSMCs. Ajoene and MATS down-regulate the activities of protein farnesyltransferase (PFTase) and protein geranylgeranyltransferase type I (PGGTase-I), contributing to the inhibition of VSMCs proliferation (Ferri et al., 2003; Golovchenko et al., 2003). AGE reduced aortic fatty streaks and carotid intima-media thickness in cholesterol-fed New Zealand rabbits and acted by reducing tissue cholesterol accumulation and inhibiting smooth muscle proliferation. Thus, AGE may protect against the development of AS (Efendy et al., 1997).

**FIGURE 5:**
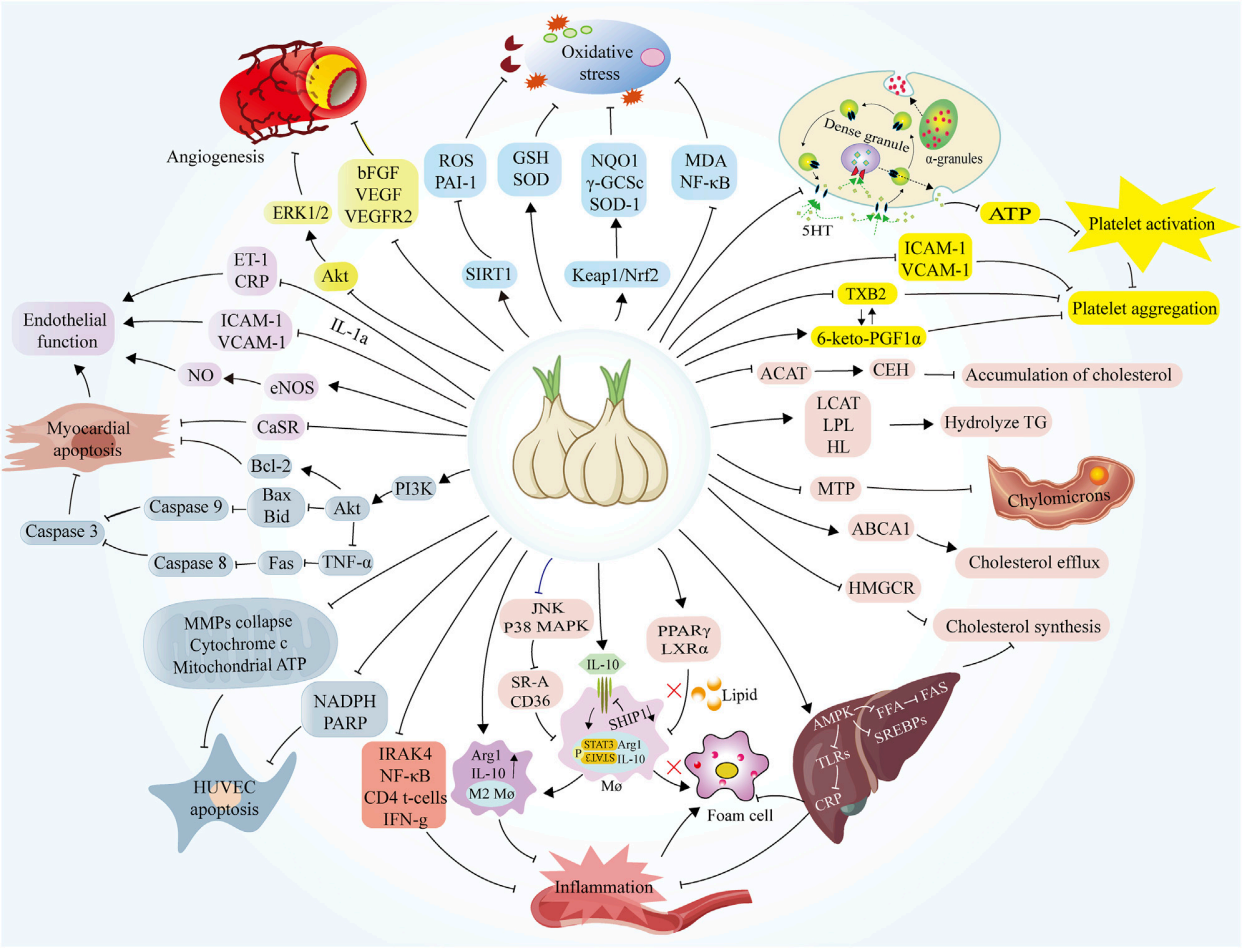
Mechanisms of the protective role of garlic in the treatment of atherosclerosis. ACAT, cholesterol acyltransferase; CEH, Cholesteryl ester hydrolase; LCAT, Lecithin cholesterol acyltransferase; LPL, Lipoprotein lipase; HL, Hepatic lipase; MTP, Microsomal triglyceride transfer protein; ABCA1, ATP Binding Cassette Subfamily A Member 1; HMGCR, Hydroxy-3-methyl glutaryl coenzyme A reductase; AMPK, AMP-activated protein kinase; FAS, Fatty acid synthase; SREBP, Sterol regulatory element-binding protein; TLR, Toll-like receptors; CRP, C-reaction protein; PPARγ, Peroxisome proliferator-activated receptor Gamma; LXRα, Liver X receptor α; IL-10, Interleukin-10; SHIP1, Src homology-2-containing inositol 5′-phosphatase 1; STAT3, Signal transducer and activator of transcription 3; Arg1, Arginase1; IRAK4, Interleukin-1 receptor-activated kinase 4; NF-κB, Nuclear factor kappa-B; IFN-g, Interferon gamma; NADPH, Nicotinamide adenine dinucleotide phosphate; PARP, Poly adenosine diphosphate-ribose polymerase; MMPs, Matrix metalloproteinases; TNF-α, Tumor necrosis factor-α; Fas, Fatty acid synthase; Bax, BCL2-Associated X; Bid, Recombinant Human BH3-Interacting Domain Death Agonist; Bcl-2, B-cell lymphoma-2; CaSR, Calcium-sensing receptor; eNOS, Endothelin nitric oxide synthase; ICAM-1, Intercellular cell adhesion molecule-1; VCAM-1, Vascular Cell Adhesion Molecule 1; ERK1/2, Extracellular regulated protein kinases; bFGF, Basic fibroblast growth factor; VEGF, Vascular endothelial growth factor; SIRT1, Sirtuin 1; ROS, Reactive oxygen species; PAI-1, Plasminogen Activator Inhibitor-1; GSH, Glutathione; SOD, Superoxide Dismutase; NQO1, NAD(P)H:quinone oxidoreductase 1; γ-GCSc, γ -glutamylcysteine synthetase antibody; SOD-1, Superoxide Dismutase 1; MDA, Malondialdehyde; TXB2, Thromboxane B2.

AS is strongly associated with the gut microbiota and its metabolites. A study confirmed that certain beneficial and anti-inflammatory gut commensal bacteria, including Faecalibacterium prausnitzii and Akkermansia spp., were significantly enriched after the 1-week allicin intervention in High-TMAO patients (Panyod et al., 2022). In diet-induced obese (DIO) mice alliin regulates glucose metabolism by reducing Lachnospiraceae and increasing Ruminococcaceae in the intestine, thereby delaying the progression of AS (Zhai et al., 2018). Garlic may be an essential prebiotic, which can induce the growth of beneficial flora (The cardiovascular effects are shown in Table 1 and Figure 5).

**TABLE 1:** Major effects and targets of garlic in atherosclerosis.

Effects	Materials or bioactive compound	Species/exposure subjects	Targets	Dose/concentration (route of administration)	Duration	Adverse effect	Reference
Hypolipidemic	Allicin	High-fat diet-induced Apo E^−/−^ mices (6 w) and peritoneal macrophage	SR-A↓	10, 20 mg/kg/d (i.g., *n* = 10)	12 w	N	Wang H et al. (2017)
			CD36↓				
			JNK↓				
			P38 MAPK↓				
	NAC	High-fat diet-induced C57BL/6 mices (3—4 w)	SREBP-1c↓	1 g/L (i.g., *n* = 15)	4 w	N	Lin and Yin, (2008)
	SEC		SREBP-2↓				
	SPC						
	SAC	FFA mixture induced HepG2 cells	AMPK↑	0.5–10 mM	24 h	N	Hwang et al. (2013)
			SREBP-1↓				
	Allicin	Ox-LDL induced THP-1 macrophage-derived foam cells	ABCA1 ↑	2.5, 5, 10, 20 and 40 g/L	3, 6, 12, 24, 48 h	N	Lin et al. (2017)
			PPARγ/LXRα ↑				
	Allicin	High-fat diet-induced Kunming mices (20 ± 2 g)	LCAT↑	1, 2, 3 mg/kg/d (i.g., *n* = 10)	10 days	N	Zhang et al. (2007)
			LPL↑				
			HL↑				
			HMGCR↓				
	FGE	HepG2	MTP↓	3, 6, 5 g/kg·bw (i.g., *n* = 6)	3, 6 h	N	Lin et al. (2002)
		Caco-2 cells					
		Male SD rats (190–210 g)					
	GPE	Human aortic subendothelial intimal smooth muscle cells from men who died of myocardial infarction	ACAT↓	allicin (3.58 mmol/L) and ajoene (0.184 mmol/L)	24 h	N	Orekhov and Tertov, (1997)
			CEH↑				
	DADS	High-fat diet-induced Wistar rats (200 ± 20 g)	SREBP-1c↓	15, 30, 60 mg/kg·bw (i.g., *n* = 10)	5 w	N	Wang et al. (2019)
			PPAR-α↑				
Antioxidant	DADS	High-fat diet-induced Wistar rats (200 ± 20 g)	Keap1/Nrf2↑	15, 30, 60 mg/kg·bw (i.g., *n* = 10)	5 w	N	Wang et al. (2019)
			NQO1↑				
			γ-GCSc↑				
			SOD-1↑				
	Allicin	H_2_O_2_ induced HUVECs	SIRT1↑	5 ng/ml	24 h	N	Hu et al. (2016)
			ROS↓				
			PAI-1↓				
	TSG	Combination of high-fat feeding, intraperitoneal injection of vitamin D3, and ovalbumin-induced inflammation in SD rats (200 ± 20 g)	MDA↓	0.6, 1.2, 2.4 g/kg/d (i.g., *n* = 12)	4 w	N	Miao et al. (2020)
			SOD↑				
	SAC	HUVECs and murine J774 macrophage cell line stimulated with ox-LDL	ROS↓	0,2.5,5,10,20 mM	1, 2, 3 h	N	Ho et al. (2001)
			NF-κB↓				
	Allicin	High cholesterol diet-induced male New Zealand rabbits (1.30 ± 0.40 kg)	MDA↓	10 mg/kg/d (po, *n* = 8)	4 w	N	El-Sheakh et al. (2016)
			SOD↑				
			GSH↑				
Antithrombotic	TSG.	Combination of high-fat feeding, intraperitoneal injection of vitamin D3, and ovalbumin-induced inflammation in SD rats (200 ± 20 g)	TXB2↓	0.6, 1.2, 2.4 g/kg/d (i.g., *n* = 12)	4 w	N	Miao et al. (2020)
			6-keto-PGF1α↑				
	Fermented garlic	Hypercholesterolemic diet-induced SD rats (180–200 g)	α-granules↓	300 mg/kg/d (po, *n* = 5)	30 days	N	Irfan et al. (2019)
			Dense granules↓				
	Garlic powder	Saline, collagen, and epinephrine induced SD rats (5 w)	VCAM-1↓	500 mg/kg·bw (i.g., *n* = 8)	7 days	N	Kim et al. (2018)
			ICAM-1↓				
	DATS	TNF-α stimulated HUVECs	JNK↓	50 or 100 μm	30 min	N	Okue et al. (2022)
			TF↓				
Inhibit angiogenesis	DATS	VEGF induced HUVECs	VEGF↓	5, 10, 20 μm	24, 48, 72 h	N	Xiao et al. (2006)
			VEGFR2↓				
			Akt ↓				
			ERK1/2 ↑				
	Allicin	Type I collagen-induced rat aorta ring	VEGF↓	0.2, 0.5, 1 m	6 days	N	Sela et al. (2008)
			bFGF↓				
	Allicin	Bovine aortic endothelial cells	VEGF↓	0.1, 0.2, 0.5, 1, 10 mm,	24 h	N	Sela et al. (2008)
			bFGF↓				
			Akt ↓				
Endothelium Protection	Allicin capsule	Elderly myocardial infarction patients (57.86 ± 11.20 a)	ET-1↓	120 mg/d (po., *n* = 75)	1 a	N	Wang and Fu, (2009)
			CRP↓				
			NO↑				
	ASE	IL-1a induced HCAECs	ICAM-1↓	0.25–4.0 mg/ml	4 days	N	Rassoul et al. (2006)
		Monocytic U937 cell line	VCAM-1↓				
	Allicin capsule	Patients with coronary heart disease combined with diabetes (61 ± 11 a)	NO↑	120 mg/d (po., *n* = 60)	90 days	N	Nie et al. (2013)
			ICAM-1↓				
	AGE	Endothelium-denuded aortic rings	eNOS↑	0.5% (w/v)	10 min	N	Takashima et al. (2017)
			NO↑				
	Allicin	Patients with angina pectoris (60.8 ± 10.7 a)	eNOS↑	60 mg/d (po., *n* = 43)	10 days	N	Nie et al. (2002)
			NO↑				
	DAT.	High glucose-induced HUVECs	MDA↓	25, 50, 100 mmol/L	24 h	N	Liu et al. (2014)
			ROS↓				
			SOD↑				
			GSH-Px↑				
	Allicin	High-fat diet induced SD rats (220—250 g)	CaSR↓	20 mg/kg/d (i.g., *n* = 12)	14 days	N	Xu et al. (2020)
	S1PC	TNF-α Induced HUVECs	GEF-H1/RhoA/Rac↓	75–300 μM	10, 15, 20, 30, 40 min, 1, 3 or 24 h	N	Kunimura et al. (2021)
	SAC		MLC kinase↓	300 μM			
	SAMC			300 μM			
Anti-Inflammatory	AGE	CE-2 diet-induced ApoE-KO mices (5 w)	CRP↓	Liquid AGE was mixed with CE-2 to make the solid content of AGE to 3% (po)	12 w	N	Morihara et al. (2017)
			TXB2↓				
			TNF-α↓				
			IRAK4↓				
			AMPK↑				
	AGE.	CE-2 diet-induced TSOD mices (4 w)	AMPK↑	the CE-2 diet containing 2% (w/w) AGE (po)	19 w	N	Miki et al. (2017)
			TLR signal path↓				
	Z-ajoene	L.P.S. induced RAW264.7	STAT3↓	10 μL	24 h	N	Hitchcock et al. (2021)
			COX2↓				
			IL1β↓				
			IL6↓				
			IL12β↓				
			IL10↑				
	Single garlic oil	High-fat diet-induced Balb/C mices (38 ± 5 g)	CD4 t-cells↓ NF-κB↓	12.5, 25, 50 mg/kg·bw/d (po., *n* = 4)	4 w	N	Lestari et al. (2020)
			TNF-a↓				
			IFN-g↓				
	AGE	C57BL/6 J, Apo E^−/−^ mices (6 w)	Arg1↑	Standard diets with or with 3% AGE (po., *n* = 5–8)	17 w	N	Miki et al. (2021)
			IL-10↑				
			CD206↑				
			HIF2α↑				
			CD68↓				
			HIF1α↓				
			IL-10rα^Ⓟ^↑				
			STAT3^Ⓟ^↑				
			SHIP1↓				
Anti-apoptotic	Allicin	ox-LDL induced HUVECs	Caspase3↓	10–100 μM	24 h	N	Chen et al. (2016)
			NADPH↓				
	Allicin	LPS induced HUVECs	MMPs collapse↓	0–40 μg/ml	24 h	N	Zhang et al. (2017)
			Cytochrome c↓				
	Allicin	H_2_O_2_ induced HUVECs	Pro-Caspase-3 Protein↓	1, 10, 20, 40 μg/ml	6, 12, 24 h	N	Chen et al. (2014)
			PARP↓				
			Bax↓				
			SOD↑				
			NO↑				
			eNOS↑				
			MDA↓				
	Garlic oil	High cholesterol diet-induced Golden Syrian hamsters (145—170 g)	PI3K-Akt↑	2% cholesterol and 1% garlic oil (po., *n* = 8)	8 w	N	Cheng et al. (2013)
			TNF-α↓				
			Fas↓				
			caspase 8, 9, 3↓				
			Bcl-2↑				
			Bid↓				
			Bax↓				
Inhibit smooth muscle proliferation	MATs	Insulin or PDGF induced VSMCs	PGGTase-I↓	50 mol/L	24 h	N	Golovchenko et al. (2003)
	AGE.	Cholesterol supplemented standard diet-induced New Zealand rabbits with carotid intima thickening (3–4 m)	PDGF↓	800 μl/kg·bw/d (po., *n* = 6)	6 w	N	Efendy et al. (1997)
Regulating gut microbiota	Alliin	High-fat diet-induced C57BL/6J mices (8 w)	Lachnospiraceae↓	0.1 mg/ml (po., *n* = 20)	8 w	N	Zhai et al. (2018)
			Ruminococcaceae↑				
	Allicin	High-TMAO patients	Prausnitzii↑	0.89 mg/ml (po., *n* = 7)	1 w	N	Panyod et al. (2022)
			Akkermansia spp↑				

The risk of AS progression is significantly increased in postmenopausal women, semmingly retale to decline level of estrogen secretion. The use of the dietary supplement “Karinat”, which is isoflavonoid-rich preparation containing tannins from garlic powder and other herbs, was proved to decrease total cholesterol by 6.3% and lower carotid intima-media thickness progression in postmenopausal women (Myasoedova et al., 2016).

## Pharmacokinetic studies of garlic

Few pharmacokinetic studies of garlic have been conducted, mainly focusing on alliin, SAC, and DATS. Three groups of rats received 8 mg/kg of alliin and allicin. The absorption and elimination of alliin radioactivity were significantly faster than other garlic components, reaching maximum blood levels within the first 10 min and almost eliminated from the blood after 6 h. Allicin did not reach maximum blood levels until 30–60 min and still existed at the end of the study after 72 h with blood levels >1000 ng-Eq/ml. The mean total urinary and fecal excretion of Allicin after 72 h was 85.5% of the dose, with urinary excretion indicating a minimum absorption rate of 65% (Perez et al., 2014). After injecting 10 mg DATS into the jugular vein of rats, the plasma concentration of DATS reached the peak of 31 μm within 1 min and gradually returned to the baseline level within 24 h. DATS was injected intravenously into rats with microemulsion, and the plasma concentration of DATS reached its peak within 3 h. Following the ingestion of DATS by human subjects, the breakdown product Allyl methyl sulfide (AMS) concentration increased to a peak at 5 h. Furthermore, following ingestion of raw garlic, AMS, allyl methyl disulfide (AMDS), DAS, DADS, DATS, and dimethyl sulfide were detected in the volunteers’ breath. The concentrations of AMDS, DAS, DADS, and DATS peaked within 2–3 h, while the concentrations of the other compounds increased more slowly (Morihara et al., 2017). Further pharmacokinetic studies of garlic are needed to determine its potential to treat AS.

## Adverse reactions and toxicity

Although garlic is generally considered safe for humans, it can still cause adverse reactions in sensitive individuals when ingested at high doses. A randomized controlled trial was conducted in which ingestion of high doses of raw garlic on an empty stomach caused changes in the intestinal flora, flatulence, and gastrointestinal disturbances to assess the safety of garlic (Rana et al., 2011). In addition, blisters, dermatitis, and burns can be observed by topical application of raw garlic (Piasek et al., 2009). *In vivo* experiments have shown that prolonged intake of high doses of raw garlic can lead to weight loss and hemolytic anemia. In addition, chronic administration of 50 mg of garlic powder per day produces anti-androgenic effects, leading to reduced sialic acid concentrations in the seminal vesicles, testes, and epididymis and reduced interstitial cell function (Rana et al., 2011). The primary toxicological mechanism of sulfide in garlic is oxidative hemolysis, which is characterized by methemoglobinemia and the formation of Heinz bodies. Early clinical signs include depression, vomiting, loss of appetite, abdominal pain, diarrhea, pale mucous membranes of the fundus, jaundice, increased heart and respiratory rates, weakness, and hemoglobinuria (Salgado et al., 2011). A low dose of garlic is safe, therapeutic dose may cause mild gastrointestinal disorder, while a high dose of garlic may cause liver damage (Rahman et al., 2016). The antithrombotic activity of garlic may interact with oral anticoagulants, so care must be taken when used in concert with oral anticoagulants (Rose et al., 1990). Allicin is a membrane-permeable compound that readily enters cells and interacts with sulfhydryl-containing compounds in cells, such as GSH or cysteine residues in proteins and enzymes containing active cysteine, resulting in cytotoxicity of allicin.

## Quality control of garlic

The quality standards of garlic or related species are included in the United States Pharmacopoeia, the European Pharmacopoeia, and the British Pharmacopoeia. The quality standards of garlic were included in the 1977 edition of the Chinese Pharmacopoeia. However, the quality standard for garlic was not found in subsequent editions of the Pharmacopoeia until it was reintroduced in the 2010 edition of the Chinese Pharmacopoeia. Since thiosulfinate and decomposition products are biologically active, and the primary precursor substance is alliin, the leading testing indexes for garlic and garlic-related species internationally are the content of alliin, the activity of allinase or the content of potential allicin. Other indicators are tested for garlic preparations prepared for various purposes and methods. The British, American, and European Pharmacopoeias all contain garlic or related preparations, with alliin or potential allicin as the leading indicator for product quality control. The United States Pharmacopoeia contains the most varieties of garlic, while the British and European Pharmacopoeia only contains garlic powder. Garlic oil is extracted from crushed garlic and includes only the fat-soluble sulfide DAS, DADS, DATS, etc., after the decomposition of allicin, but no water-soluble components and alliin. Garlic extract is extracted by organic solvent, which inhibits allinase activity, and the extract consists of fat-soluble sulfide and allicin without allicin. The preparation prepared by pulverizing garlic cloves into powder contains alliin and a small amount of fat-soluble sulfide (Harauma and Moriguchi, 2006; Corzo-Martínez et al., 2007). Freeze-dried garlic powder and its preparations made from garlic by low-temperature freeze-drying can produce allicin under suitable conditions *in vivo* due to the retention of alliin and active allinase. Therefore the European Pharmacopoeia and the United States Pharmacopoeia require determining the potential allicin content for the corresponding preparations and raw materials.

## Conclusion and perspective

The antiatherogenic effects and mechanisms of garlic were discussed in this review, and it was thought that further research should be conducted in the future on the following aspects. Garlic contains multi-bioactive components, such as allicin, DAS, DADS, and DATS, among which allicin is the primary bioactive substance of garlic. DATS has been included in the Chinese Pharmacopoeia (Chinese Pharmacopoeia Commission, 2015), and alliin and allicin have been included in the European Pharmacopoeia (European Pharmacopoeia, 2017). The research progress of Allicin is restricted because of its unstable chemical properties and difficulties in preparation and storage. China has established a patented technology for preparing Allicin, which can extract more stable and higher purity allicin, making it possible to conduct in-depth research. Therefore, there is an urgent need to conduct pharmacological studies on garlic and its active ingredients in the future to clarify the actual active ingredients of garlic as soon as possible.

Recent studies have shown that whole garlic or its components/extracts exhibit multiple preventive and therapeutic effects on AS. The formation of AS is a multifactorial interaction, so it should be studied in depth from various pathways, links, and targets. The biological effects of garlic may meet the need for a multi-targeted therapeutic strategy for AS. However, some contradictory results may be related to the inconsistency between the quality, extraction, preparation, and dosage of garlic and the experimental objects and methods. Therefore, future research on garlic should be deepened in the above aspects. Garlic may exert antiatherogenic effects through hypolipidemic, antioxidant, antithrombotic, inhibiting angiogenesis, protecting endothelial cells, anti-inflammatory, anti-apoptotic, inhibiting vascular smooth muscle proliferation, and regulating gut microbiota. However, the potential mechanisms of absorption, distribution, metabolism, and excretion of garlic and its components/extracts and the synergistic or antagonistic effects between components are unknown, for which further studies should be conducted.

Garlic can treat AS by regulating different signaling pathways, such as AMPK/TLRs, Keap1/Nrf2, PI3K/AKT, PPARγ/LXRα, GEF-H1/RhoA/Rac, etc. However, there is still no molecular mechanism for clinical AS patients. Therefore, the direct AS protection mechanisms of garlic have not been explored. Further studies in animals and humans should evaluate the protective ability of single garlic-derived compounds against AS. Future studies should also focus on the beneficial effects of whole garlic and garlic-derived compounds on AS based on relevant signaling pathways. There have been few clinical trials to monitor garlic’s therapeutic effect in recent years. Therefore, there is an urgent need for large randomized, controlled, and double-blind trials to assess the efficacy and safety of garlic in the treatment of AS from the perspective of clinical practice. In addition, it will make the clinical application of garlic safer and more effective in solving the adverse reactions of garlic by inhibiting oxidized hemolysate and reducing the risk of bleeding.

## Glossary

5-HT: 5-hydroxy tryptamine
6-keto-PGF1α: 6-keto-prostaglandin F1α
AA: Arachidonic acid
ABCA1: ATP Binding Cassette Subfamily A Member 1
ACAT: Acyl-CoA:cholesterol acyltransferase
ACE: Angiotensin-Converting Enzyme
AGE: Aged garlic extract
AMPK: AMP-activated protein kinase
APTT: Activated partial thromboplasting time
Arg1: Arginase1
AS: Atherosclerosis
Bax: BCL2-Associated X
Bcl-2: B-cell lymphoma-2
bFGF: Basic fibroblast growth factor
Bid: Recombinant Human BH3-Interacting Domain Death Agonist
CAM: Complementary and alternate medicine
CaMKK: Calcium/calmodulin-dependent kinase kinase
CaSR: Calcium-sensing receptor
CD36: Cluster of differentiation 36
CEH: Cholesteryl ester hydrolase
cGMP: Cyclic guanosine monophosphate
CHD: Coronary artery heart disease
COX1: Cyclooxygenase-1
COX2: Cyclooxygenase-2
CRP: C-reaction protein
CVDs: Cardiovascular diseases
DADS: Diallyl disulfide
DAS: Diallyl sulfide
DATS: Diallyl trisulfide
DATTS: Diallyl Tetrasulfide
DIO: Diet-induced obese
ECs: Endothelial cells
EDHF: Endothelium-derived hyperpolarizing factor
ELAM-1: Endothelial leukocyte adhesion molecule-1
eNOS: Endothelin nitric oxide synthase
ERK1/2: Extracellular regulated protein kinases
ET1: Endothelin-1
FAS: Fatty acid synthase
FGE: Fresh garlic extract
FMD: Flow-mediated dilation
G6PDH: Glucose-6-Phosphate Dehydrogenase
GEF-H1: Guanine nucleotide exchange factor-H1
GMP140: Granular membrane protein 140
GPE: Garlic powder extract
GSH: Glutathione
GSH-Px: Glutathione peroxidase
HDL: High-density lipoprotein
HIF2α: Hypoxia-inducible factor 2α
HL: Hepatic lipase
HMGCR: Hydroxy-3-methyl glutaryl coenzyme A reductase
hsCRP: High-sensitivity C-reactive protein
HUVECs: Human umbilical vein endothelial cells
ICAM-1: Intercellular cell adhesion molecule-1
IFN-g: Interferon gamma
IL-10: Interleukin-10
IL-10Rα: IL-10 receptor α
IL-12β: Interleukin-12 beta
IL-18: Interleukin 18
IL-1a: Interleukin-1 alpha
IL-1β: Interleukin-1 beta
IL-6: Interleukin- 6
IL-8: Interleukin-8
iNOS: Inducible nitric oxide synthase
IRAK4: Interleukin-1 receptor-activated kinase 4
JAK3: Janus Kinase 3
JNK: C-Jun N-terminal kinase
LCAT: Lecithin cholesterol acyltransferase
LDL: Low-density lipoprotein
LDL-C: Low-density lipoprotein cholesterol
L-NAME: L-N G-Nitro arginine methyl ester
LPL: Lipoprotein lipase
LPS: Lipopolysaccharide
LTC4C: Leukotriene C4
LXRα: Liver X receptor α
MAPK: Mitogen-activated protein kinase
MATS: Methyl allyl trisulfide
MCP-1: Monocyte chemotactic protein 1
MDA: Malondialdehyde
MLC: Myosin light chain
MMPs: Matrix metalloproteinases
MTTP: Microsomal triglyceride transfer protein
Mø: Macrophages
NAC: N-acetylcysteine
NADPH: Nicotinamide adenine dinucleotide phosphate
NF-κB: Nuclear factor kappa-B
NO: Nitric oxide
NOS: Nitric oxide synthase
NQO1: NAD(P)H:quinone oxidoreductase 1
Nrf2: Nuclear erythroid 2-related factor 2
OS: Oxidative stress
ox-LDL: LDL oxidation
p38 MAPK: P38 mitogen-activated protein kinase
PAI-1: Plasminogen Activator Inhibitor-1
PARP: Poly adenosine diphosphate-ribose polymerase
PDGF: Platelet-Derived Growth Factor
PGE2: Prostaglandin E2
PGGTase-I: Protein geranylgeranyltransferase type I
PGI2: Prostaglandin I2
PPARα: Peroxisome proliferator-activated receptor alpha
PPARγ: Peroxisome proliferator-activated receptor Gamma
RhoA: Ras homolog gene family, member A
RNS: Reactive nitrogen species
ROS: Reactive oxygen species
S1PC: S-1-propenylcysteine
SAC: S-allyl cysteine
SACS: S-allyl cysteine sulfoxide
SEC: S-acetylcysteine
SHIP1: Src homology-2-containing inositol 5′-phosphatase 1
SI: Stiffness index
SIRT1: Sirtuin 1
STAT3: Signal transducer and activator of transcription 3
SOD: Superoxide Dismutase
SOD-1: Superoxide Dismutase 1
SPC: S-propylcysteine
SR-A: Scavenger receptor class A
SREBP-1: Sterol regulatory element-binding proteins-1
SREBP-1c: Sterol regulatory element-binding protein-1c
SREBP-2: Sterol regulatory element-binding protein-2
T-AOC: Total antioxidant capacit
TAS: Total antioxidant status
TC: Total cholesterol
TF: Tissue factor
Th1: Thelper 1 cell
TLR: Toll-like receptors
TNF-α: Tumor necrosis factor-α
TSG: Total Saponins of Garlic
TSOD: Tsumura Suzuki Obese Diabetes
TXA2: Thromboxane A2
TXB2: Thromboxane B2
VCAM-1: Vascular Cell Adhesion Molecule 1
VEGF: Vascular endothelial growth factor
VEGF R2: Vascular endothelial growth factor receptor 2
VSMCs: Vascular smooth muscle cells
γ-GCSc: γ -glutamylcysteine synthetase antibody
