# Supplementary material for: Roles and mechanisms of garlic and its extracts on atherosclerosis: A review
Source: Front Pharmacol. 2022 Oct 3;13:954938. doi: 10.3389/fphar.2022.954938 (PMC9574545; doi:10.3389/fphar.2022.954938)
Supplement: Supplementary file 1 [file DataSheet1.doc]

**Appendix 1. Search Syntaxes**

**Table 1**

**PubMed**

**A search performed on: 1-5-2022**

**Total number of results: 903**

| Search number | Search Details | Results |
| --- | --- | --- |
| 24 | (("Garlic"[MeSH Terms] OR ("allium sativum"[Title/Abstract] OR "Allicin"[Title/Abstract] OR "Knoblauch"[Title/Abstract] OR "garlicin"[Title/Abstract] OR "garlic oil"[Title/Abstract] OR (("Garlic"[MeSH Terms] OR "Garlic"[All Fields] OR "garlic s"[All Fields] OR "garlics"[All Fields]) AND "oil macerates"[Title/Abstract]) OR "garlic extract"[Title/Abstract] OR "garlic cloves"[Title/Abstract] OR "garlic powder"[Title/Abstract] OR "aged garlic extract"[Title/Abstract] OR "alliinase"[Title/Abstract])) AND ("Cholesterol"[MeSH Terms] OR "Epicholesterol"[Title/Abstract] OR "Hyperlipidemias"[MeSH Terms] OR ("lipemia*"[Title/Abstract] OR "lipidemia*"[Title/Abstract] OR "hyperlipemia*"[Title/Abstract] OR "lipid"[Title/Abstract] OR "Hypercholesterolaemias"[Title/Abstract] OR ((("Cholesterol"[MeSH Terms] OR "Cholesterol"[All Fields] OR "cholesterol s"[All Fields] OR "cholesterole"[All Fields] OR "Cholesterols"[All Fields]) AND "level*"[All Fields]) AND "High"[Title/Abstract]) OR "high cholesterol level*"[Title/Abstract] OR ("level*"[All Fields] AND "high cholesterol"[Title/Abstract]) OR "elevated cholesterol"[Title/Abstract] OR ("cholesterol*"[All Fields] AND "Elevated"[Title/Abstract]) OR "elevated cholesterols"[Title/Abstract] OR "hypercholesteremia*"[Title/Abstract] OR "Hyperlipidaemia"[Title/Abstract] OR "Dyslipidaemia"[Title/Abstract]))) OR (("Garlic"[MeSH Terms] OR ("allium sativum"[Title/Abstract] OR "Allicin"[Title/Abstract] OR "Knoblauch"[Title/Abstract] OR "garlicin"[Title/Abstract] OR "garlic oil"[Title/Abstract] OR (("Garlic"[MeSH Terms] OR "Garlic"[All Fields] OR "garlic s"[All Fields] OR "garlics"[All Fields]) AND "oil macerates"[Title/Abstract]) OR "garlic extract"[Title/Abstract] OR "garlic cloves"[Title/Abstract] OR "garlic powder"[Title/Abstract] OR "aged garlic extract"[Title/Abstract] OR "alliinase"[Title/Abstract])) AND ("Atheroscleroses"[Title/Abstract] OR "Atherogenesis"[Title/Abstract] OR "Atherosclerosis"[MeSH Terms])) OR (("Garlic"[MeSH Terms] OR ("allium sativum"[Title/Abstract] OR "Allicin"[Title/Abstract] OR "Knoblauch"[Title/Abstract] OR "garlicin"[Title/Abstract] OR "garlic oil"[Title/Abstract] OR (("Garlic"[MeSH Terms] OR "Garlic"[All Fields] OR "garlic s"[All Fields] OR "garlics"[All Fields]) AND "oil macerates"[Title/Abstract]) OR "garlic extract"[Title/Abstract] OR "garlic cloves"[Title/Abstract] OR "garlic powder"[Title/Abstract] OR "aged garlic extract"[Title/Abstract] OR "alliinase"[Title/Abstract])) AND ("cardiovascular diseases"[Title/Abstract] OR "Angiocardiopathy"[Title/Abstract] OR "cardiovascular disorders"[Title/Abstract] OR "cardiology disease"[Title/Abstract] OR "cardiovascular disease"[Title/Abstract])) OR (("Garlic"[MeSH Terms] OR ("allium sativum"[Title/Abstract] OR "Allicin"[Title/Abstract] OR "Knoblauch"[Title/Abstract] OR "garlicin"[Title/Abstract] OR "garlic oil"[Title/Abstract] OR (("Garlic"[MeSH Terms] OR "Garlic"[All Fields] OR "garlic s"[All Fields] OR "garlics"[All Fields]) AND "oil macerates"[Title/Abstract]) OR "garlic extract"[Title/Abstract] OR "garlic cloves"[Title/Abstract] OR "garlic powder"[Title/Abstract] OR "aged garlic extract"[Title/Abstract] OR "alliinase"[Title/Abstract])) AND ("Coronary Disease"[MeSH Terms] OR (("disease*"[All Fields] AND "Coronary"[Title/Abstract]) OR "coronary heart disease*"[Title/Abstract] OR ("disease*"[All Fields] AND "coronary heart"[Title/Abstract]) OR ((("Heart"[MeSH Terms] OR "Heart"[All Fields] OR "hearts"[All Fields] OR "heart s"[All Fields]) AND "disease*"[All Fields]) AND "Coronary"[Title/Abstract])))) OR (("Garlic"[MeSH Terms] OR ("allium sativum"[Title/Abstract] OR "Allicin"[Title/Abstract] OR "Knoblauch"[Title/Abstract] OR "garlicin"[Title/Abstract] OR "garlic oil"[Title/Abstract] OR (("Garlic"[MeSH Terms] OR "Garlic"[All Fields] OR "garlic s"[All Fields] OR "garlics"[All Fields]) AND "oil macerates"[Title/Abstract]) OR "garlic extract"[Title/Abstract] OR "garlic cloves"[Title/Abstract] OR "garlic powder"[Title/Abstract] OR "aged garlic extract"[Title/Abstract] OR "alliinase"[Title/Abstract])) AND ("Angina Pectoris"[MeSH Terms] OR ("Stenocardia"[Title/Abstract] OR "Stenocardias"[Title/Abstract] OR "angor pectoris"[Title/Abstract]))) | 903 |
| 23 | ("Garlic"[MeSH Terms] OR ("allium sativum"[Title/Abstract] OR "Allicin"[Title/Abstract] OR "Knoblauch"[Title/Abstract] OR "garlicin"[Title/Abstract] OR "garlic oil"[Title/Abstract] OR (("Garlic"[MeSH Terms] OR "Garlic"[All Fields] OR "garlic s"[All Fields] OR "garlics"[All Fields]) AND "oil macerates"[Title/Abstract]) OR "garlic extract"[Title/Abstract] OR "garlic cloves"[Title/Abstract] OR "garlic powder"[Title/Abstract] OR "aged garlic extract"[Title/Abstract] OR "alliinase"[Title/Abstract])) AND ("Angina Pectoris"[MeSH Terms] OR ("Stenocardia"[Title/Abstract] OR "Stenocardias"[Title/Abstract] OR "angor pectoris"[Title/Abstract])) | 3 |
| 22 | ("Garlic"[MeSH Terms] OR ("allium sativum"[Title/Abstract] OR "Allicin"[Title/Abstract] OR "Knoblauch"[Title/Abstract] OR "garlicin"[Title/Abstract] OR "garlic oil"[Title/Abstract] OR (("Garlic"[MeSH Terms] OR "Garlic"[All Fields] OR "garlic s"[All Fields] OR "garlics"[All Fields]) AND "oil macerates"[Title/Abstract]) OR "garlic extract"[Title/Abstract] OR "garlic cloves"[Title/Abstract] OR "garlic powder"[Title/Abstract] OR "aged garlic extract"[Title/Abstract] OR "alliinase"[Title/Abstract])) AND ("Coronary Disease"[MeSH Terms] OR (("disease*"[All Fields] AND "Coronary"[Title/Abstract]) OR "coronary heart disease*"[Title/Abstract] OR ("disease*"[All Fields] AND "coronary heart"[Title/Abstract]) OR ((("Heart"[MeSH Terms] OR "Heart"[All Fields] OR "hearts"[All Fields] OR "heart s"[All Fields]) AND "disease*"[All Fields]) AND "Coronary"[Title/Abstract]))) | 91 |
| 21 | ("Garlic"[MeSH Terms] OR ("allium sativum"[Title/Abstract] OR "Allicin"[Title/Abstract] OR "Knoblauch"[Title/Abstract] OR "garlicin"[Title/Abstract] OR "garlic oil"[Title/Abstract] OR (("Garlic"[MeSH Terms] OR "Garlic"[All Fields] OR "garlic s"[All Fields] OR "garlics"[All Fields]) AND "oil macerates"[Title/Abstract]) OR "garlic extract"[Title/Abstract] OR "garlic cloves"[Title/Abstract] OR "garlic powder"[Title/Abstract] OR "aged garlic extract"[Title/Abstract] OR "alliinase"[Title/Abstract])) AND ("cardiovascular diseases"[Title/Abstract] OR "Angiocardiopathy"[Title/Abstract] OR "cardiovascular disorders"[Title/Abstract] OR "cardiology disease"[Title/Abstract] OR "cardiovascular disease"[Title/Abstract]) | 174 |
| 20 | ("Garlic"[MeSH Terms] OR ("allium sativum"[Title/Abstract] OR "Allicin"[Title/Abstract] OR "Knoblauch"[Title/Abstract] OR "garlicin"[Title/Abstract] OR "garlic oil"[Title/Abstract] OR (("Garlic"[MeSH Terms] OR "Garlic"[All Fields] OR "garlic s"[All Fields] OR "garlics"[All Fields]) AND "oil macerates"[Title/Abstract]) OR "garlic extract"[Title/Abstract] OR "garlic cloves"[Title/Abstract] OR "garlic powder"[Title/Abstract] OR "aged garlic extract"[Title/Abstract] OR "alliinase"[Title/Abstract])) AND ("Atheroscleroses"[Title/Abstract] OR "Atherogenesis"[Title/Abstract] OR "Atherosclerosis"[MeSH Terms]) | 38 |
| 19 | ("Garlic"[MeSH Terms] OR ("allium sativum"[Title/Abstract] OR "Allicin"[Title/Abstract] OR "Knoblauch"[Title/Abstract] OR "garlicin"[Title/Abstract] OR "garlic oil"[Title/Abstract] OR (("Garlic"[MeSH Terms] OR "Garlic"[All Fields] OR "garlic s"[All Fields] OR "garlics"[All Fields]) AND "oil macerates"[Title/Abstract]) OR "garlic extract"[Title/Abstract] OR "garlic cloves"[Title/Abstract] OR "garlic powder"[Title/Abstract] OR "aged garlic extract"[Title/Abstract] OR "alliinase"[Title/Abstract])) AND ("Cholesterol"[MeSH Terms] OR "Epicholesterol"[Title/Abstract] OR "Hyperlipidemias"[MeSH Terms] OR ("lipemia*"[Title/Abstract] OR "lipidemia*"[Title/Abstract] OR "hyperlipemia*"[Title/Abstract] OR "lipid"[Title/Abstract] OR "Hypercholesterolaemias"[Title/Abstract] OR ((("Cholesterol"[MeSH Terms] OR "Cholesterol"[All Fields] OR "cholesterol s"[All Fields] OR "cholesterole"[All Fields] OR "Cholesterols"[All Fields]) AND "level*"[All Fields]) AND "High"[Title/Abstract]) OR "high cholesterol level*"[Title/Abstract] OR ("level*"[All Fields] AND "high cholesterol"[Title/Abstract]) OR "elevated cholesterol"[Title/Abstract] OR ("cholesterol*"[All Fields] AND "Elevated"[Title/Abstract]) OR "elevated cholesterols"[Title/Abstract] OR "hypercholesteremia*"[Title/Abstract] OR "Hyperlipidaemia"[Title/Abstract] OR "Dyslipidaemia"[Title/Abstract])) | 737 |
| 18 | "Angina Pectoris"[MeSH Terms] OR "Stenocardia"[Title/Abstract] OR "Stenocardias"[Title/Abstract] OR "angor pectoris"[Title/Abstract] | 44,388 |
| 17 | "Stenocardia"[Title/Abstract] OR "Stenocardias"[Title/Abstract] OR "angor pectoris"[Title/Abstract] | 954 |
| 16 | "Angina Pectoris"[MeSH Terms] | 44,234 |
| 15 | "Coronary Disease"[MeSH Terms] OR (("disease*"[All Fields] AND "Coronary"[Title/Abstract]) OR "coronary heart disease*"[Title/Abstract] OR ("disease*"[All Fields] AND "coronary heart"[Title/Abstract]) OR ((("Heart"[MeSH Terms] OR "Heart"[All Fields] OR "hearts"[All Fields] OR "heart s"[All Fields]) AND "disease*"[All Fields]) AND "Coronary"[Title/Abstract])) | 358,585 |
| 14 | ("disease*"[All Fields] AND "Coronary"[Title/Abstract]) OR "coronary heart disease*"[Title/Abstract] OR ("disease*"[All Fields] AND "coronary heart"[Title/Abstract]) OR ((("Heart"[MeSH Terms] OR "Heart"[All Fields] OR "hearts"[All Fields] OR "heart s"[All Fields]) AND "disease*"[All Fields]) AND "Coronary"[Title/Abstract]) | 283,088 |
| 13 | "Coronary Disease"[MeSH Terms] | 228,950 |
| 12 | "cardiovascular diseases"[Title/Abstract] OR "Angiocardiopathy"[Title/Abstract] OR "cardiovascular disorders"[Title/Abstract] OR "cardiology disease"[Title/Abstract] OR "cardiovascular disease"[Title/Abstract] | 213,279 |
| 11 | "Atheroscleroses"[Title/Abstract] OR "Atherogenesis"[Title/Abstract] OR "Atherosclerosis"[MeSH Terms] | 60,620 |
| 10 | "Atheroscleroses"[Title/Abstract] OR "Atherogenesis"[Title/Abstract] | 13,461 |
| 9 | "Atherosclerosis"[MeSH Terms] | 50,891 |
| 8 | "Cholesterol"[MeSH Terms] OR "Epicholesterol"[Title/Abstract] OR "Hyperlipidemias"[MeSH Terms] OR ("lipemia*"[Title/Abstract] OR "lipidemia*"[Title/Abstract] OR "hyperlipemia*"[Title/Abstract] OR "lipid"[Title/Abstract] OR "Hypercholesterolaemias"[Title/Abstract] OR ((("Cholesterol"[MeSH Terms] OR "Cholesterol"[All Fields] OR "cholesterol s"[All Fields] OR "cholesterole"[All Fields] OR "Cholesterols"[All Fields]) AND "level*"[All Fields]) AND "High"[Title/Abstract]) OR "high cholesterol level*"[Title/Abstract] OR ("level*"[All Fields] AND "high cholesterol"[Title/Abstract]) OR "elevated cholesterol"[Title/Abstract] OR ("cholesterol*"[All Fields] AND "Elevated"[Title/Abstract]) OR "elevated cholesterols"[Title/Abstract] OR "hypercholesteremia*"[Title/Abstract] OR "Hyperlipidaemia"[Title/Abstract] OR "Dyslipidaemia"[Title/Abstract]) | 640,253 |
| 7 | "lipemia*"[Title/Abstract] OR "lipidemia*"[Title/Abstract] OR "hyperlipemia*"[Title/Abstract] OR "lipid"[Title/Abstract] OR "Hypercholesterolaemias"[Title/Abstract] OR ((("Cholesterol"[MeSH Terms] OR "Cholesterol"[All Fields] OR "cholesterol s"[All Fields] OR "cholesterole"[All Fields] OR "Cholesterols"[All Fields]) AND "level*"[All Fields]) AND "High"[Title/Abstract]) OR "high cholesterol level*"[Title/Abstract] OR ("level*"[All Fields] AND "high cholesterol"[Title/Abstract]) OR "elevated cholesterol"[Title/Abstract] OR ("cholesterol*"[All Fields] AND "Elevated"[Title/Abstract]) OR "elevated cholesterols"[Title/Abstract] OR "hypercholesteremia*"[Title/Abstract] OR "Hyperlipidaemia"[Title/Abstract] OR "Dyslipidaemia"[Title/Abstract] | 520,105 |
| 6 | "Hyperlipidemias"[MeSH Terms] | 68,967 |
| 5 | "Epicholesterol"[Title/Abstract] | 85 |
| 4 | "Cholesterol"[MeSH Terms] | 167,381 |
| 3 | "Garlic"[MeSH Terms] OR ("allium sativum"[Title/Abstract] OR "Allicin"[Title/Abstract] OR "Knoblauch"[Title/Abstract] OR "garlicin"[Title/Abstract] OR "garlic oil"[Title/Abstract] OR (("Garlic"[MeSH Terms] OR "Garlic"[All Fields] OR "garlic s"[All Fields] OR "garlics"[All Fields]) AND "oil macerates"[Title/Abstract]) OR "garlic extract"[Title/Abstract] OR "garlic cloves"[Title/Abstract] OR "garlic powder"[Title/Abstract] OR "aged garlic extract"[Title/Abstract] OR "alliinase"[Title/Abstract]) | 5,690 |
| 2 | "allium sativum"[Title/Abstract] OR "Allicin"[Title/Abstract] OR "Knoblauch"[Title/Abstract] OR "garlicin"[Title/Abstract] OR "garlic oil"[Title/Abstract] OR (("garlic"[MeSH Terms] OR "garlic"[All Fields] OR "garlic s"[All Fields] OR "garlics"[All Fields]) AND "oil macerates"[Title/Abstract]) OR "garlic extract"[Title/Abstract] OR "garlic cloves"[Title/Abstract] OR "garlic powder"[Title/Abstract] OR "aged garlic extract"[Title/Abstract] OR "alliinase"[Title/Abstract] | 3,815 |
| 1 | "Garlic"[MeSH Terms] | 3,820 |

**Table 2**

**EMBASE**

**A search performed on 1-5-2022**

**Total number of results: 2374**

| No. | Query | Results |
| --- | --- | --- |
| #18 | #3 AND #17 | 2374 |
| #17 | #6 OR #9 OR #12 OR #13 OR #16 | 5332463 |
| #16 | #14 OR #15 | 109877 |
| #15 | stenocardia:ab,ti OR stenocardias:ab,ti OR 'angor pectoris':ab,ti | 946 |
| #14 | 'angina pectoris'/exp | 109701 |
| #13 | 'coronary disease*':ab,ti OR 'disease*, coronary':ab,ti OR 'coronary heart disease*':ab,ti OR 'disease*, coronary heart':ab,ti OR 'heart disease*, coronary':ab,ti | 95978 |
| #12 | #10 OR #11 | 4940618 |
| #11 | angiocardiopathy:ab,ti OR 'cardiovascular disorders':ab,ti OR 'cardiology disease':ab,ti OR 'cardiovascular disease':ab,ti | 218237 |
| #10 | 'cardiovascular disease'/exp | 4906615 |
| #9 | #7 OR #8 | 245074 |
| #8 | atheroscleroses:ab,ti OR atherogenesis:ab,ti | 17533 |
| #7 | 'atherosclerosis'/exp | 241362 |
| #6 | #4 OR #5 | 639290 |
| #5 | lipids:ab,ti OR cholesterol:ab,ti OR hyperlipidemia:ab,ti OR 'high cholesterol level':ab,ti OR 'level, high cholesterol':ab,ti OR 'levels, high cholesterol':ab,ti OR 'elevated cholesterol':ab,ti OR 'cholesterol, elevated':ab,ti OR 'cholesterols, elevated':ab,ti OR 'elevated cholesterols':ab,ti OR hypercholesteremia:ab,ti OR hypercholesteremias:ab,ti OR hyperlipidaemia:ab,ti OR dyslipidaemia:ab,ti OR hypercholesterolaemias:ab,ti OR 'high cholesterol levels':ab,ti OR 'cholesterol level, high':ab,ti OR 'cholesterol levels, high':ab,ti | 531995 |
| #4 | 'cholesterol'/exp | 357250 |
| #3 | #1 OR #2 | 10932 |
| #2 | 'allium sativum':ab,ti OR allicin:ab,ti OR knoblauch:ab,ti OR garlicin:ab,ti OR 'garlic oil':ab,ti OR 'garlic oil macerates':ab,ti OR 'garlic extract':ab,ti OR 'garlic cloves':ab,ti OR 'garlic powder':ab,ti OR 'aged garlic extract':ab,ti OR alliinase:ab,ti | 5065 |
| #1 | 'garlic'/exp | 8624 |

**Table 3**

**Web of science**

**A search performed on: 1-5-2022**

**Total number of results: 3,392**

12

**#11 OR #9 OR #7 OR #5 OR #3**

[3,392](http://www-webofscience-com-s.vpn1.hactcm.edu.cn/wos/alldb/summary/26c1106b-d85f-4182-9e52-1c2f0f3d1baf-2bfd1f45/relevance/1)

11

**#10 AND #1**

[5](http://www-webofscience-com-s.vpn1.hactcm.edu.cn/wos/alldb/summary/af42831a-2312-4809-8487-e35583d5528b-2bfd1d38/relevance/1)

10

**(((TS=(Angina Pectoris)) OR TS=(Stenocardia)) OR TS=(Stenocardias)) OR TS=(Angor Pectoris)**

[60,841](http://www-webofscience-com-s.vpn1.hactcm.edu.cn/wos/alldb/summary/efb8a48a-cba7-4adf-abce-7c99a93acde9-2bfd1b86/relevance/1)

9

**#8 AND #1**

[306](http://www-webofscience-com-s.vpn1.hactcm.edu.cn/wos/alldb/summary/81de70e5-54c1-45cd-9c32-e3ad97729f59-2bfd16a4/relevance/1)

8

**((((TS=(Coronary Disease*)) OR TS=(Disease*, Coronary)) OR TS=(Coronary Heart Disease*)) OR TS=(Disease*, Coronary Heart)) OR TS=(Heart Disease*, Coronary)**

[644,828](http://www-webofscience-com-s.vpn1.hactcm.edu.cn/wos/alldb/summary/472b3b0a-762b-46dd-89f2-bac15558c9b3-2bfd1528/relevance/1)

7

**#6 AND #1**

[1,296](http://www-webofscience-com-s.vpn1.hactcm.edu.cn/wos/alldb/summary/357f1d61-6b23-418e-8041-1627cb024aa0-2bfd0f10/relevance/1)

6

**((((TS=(Cardiovascular diseases)) OR TS=(Angiocardiopathy)) OR TS=(cardiovascular disorders)) OR TS=(cardiology disease)) OR TS=(cardiovascular disease)**

[2,354,969](http://www-webofscience-com-s.vpn1.hactcm.edu.cn/wos/alldb/summary/87115297-2701-4c9c-8937-63ff25e8e08a-2bfd0dc3/relevance/1)

5

**#4 AND #1**

[382](http://www-webofscience-com-s.vpn1.hactcm.edu.cn/wos/alldb/summary/7fdad7b2-77e1-42c5-b521-ab32d9b62c9d-2bfd059b/relevance/1)

4

**((TS=(Atherosclerosis)) OR TS=(Atheroscleroses)) OR TS=(Atherogenesis)**

[259,224](http://www-webofscience-com-s.vpn1.hactcm.edu.cn/wos/alldb/summary/aebbd32e-3c38-4b98-b63a-177caebb0f1e-2bfd038d/relevance/1)

3

**#2 AND #1**

[2,607](http://www-webofscience-com-s.vpn1.hactcm.edu.cn/wos/alldb/summary/72e67a25-d5a1-48ec-936b-5947e04efa8d-2bfcfe54/relevance/1)

2

**((((((((((((((((TS=(Cholesterol)) OR TS=(Epicholesterol)) OR TS=(Hyperlipidemia*)) OR TS=(Hyperlipemia*)) OR TS=(Lipidemia*)) OR TS=(Lipemia*)) OR TS=(lipid)) OR TS=(Hypercholesterolaemias)) OR TS=(Cholesterol Level*, High )) OR TS=(High Cholesterol Level* )) OR TS=(Level*, High Cholesterol )) OR TS=(Elevated Cholesterol)) OR TS=(Cholesterol*, Elevated )) OR TS=(Elevated Cholesterols)) OR TS=(Hypercholesteremia*)) OR TS=(Hyperlipidaemia )) OR TS=(Dyslipidaemia)**

[1,748,730](http://www-webofscience-com-s.vpn1.hactcm.edu.cn/wos/alldb/summary/46862e14-6bc5-4497-8c89-ae3194ef6692-2bfcfb81/relevance/1)

1

**(((((((((((((TS=(Garlic))) OR TS=(allium sativum))) OR TS=(Allicin)) OR TS=(Knoblauch)) OR TS=(garlicin)) OR TS=(garlic oil)) OR TS=(garlic oil macerates )) OR TS=(garlic extract )) OR TS=(garlic cloves)) OR TS=(garlic powder)) OR TS=(aged garlic extract)) OR TS=(alliinase)**
